# Methodological Challenges in the Application of QSAR Models for Chemical Prioritization and Toxicity Assessment: A Case Study on Aryl Hydrocarbon Receptor Activity in Environmental Pollutant Mixtures

**DOI:** 10.1021/acsenvironau.5c00224

**Published:** 2026-01-21

**Authors:** Jiří Komprda, Katarína Lörinczová, Zuzana Toušová, Marie Smutná, Soňa Smetanová, Klára Komprdová, Klára Hilscherová

**Affiliations:** RECETOX, Faculty of Science, 37748 Masaryk University, Kotlarska 2, 602 00 Brno, Czech Republic

**Keywords:** QSAR modeling, aryl hydrocarbon receptor, mixture toxicity, water pollutants, compound prioritization, in vitro testing

## Abstract

The complexity of chemical mixtures in the environment challenges their in-depth risk assessment due to the diverse compounds in use and the lack of experimental toxicity data. In silico models can be used to fill data gaps for compounds with unknown toxic potency. QSAR models typically distinguish only between active and inactive compounds, providing no information about the levels of activity. In this study, a quantitative structure–activity relationship (QSAR) model that classifies compounds into multiple activity levels was developed to address data gaps in the levels of aryl hydrocarbon receptor-mediated (AhR) activity of compounds commonly detected in environmental samples. Its practical applicability has been demonstrated on highly complex mixtures of aquatic pollutants from the Joined Danube Survey to prioritize the most relevant compounds for experimental assessment. The model’s performance showed high sensitivity and specificity, with weighted overall accuracy ranging from 77 to 87%. The combination of experimental and QSAR predicted data was used to calculate site-specific AhR activity, which was compared to the overall AhR activity detected by in vitro bioassays. Experimental testing confirmed the ability of the QSAR model to identify compounds with high AhR activity, including benzonaphthothiophene, perylene, acridone, and triphenylene, and prioritize the most relevant suspected effect drivers. Our model can predict toxic potency and thus prioritize the potential bioactive compounds based on specific activity levels. Our study shows that when QSAR models are used for compound prioritization, several factors must be considered: cytotoxicity, solubility, the high rate of false positives for low-toxicity compounds, and the model’s applicability domain.

## Graphical abstract

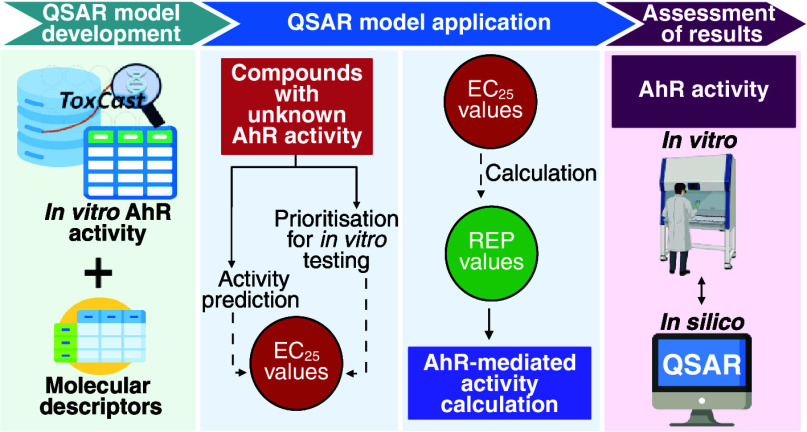

## Introduction

Complex mixtures of micropollutants in surface waters, primarily originating from anthropogenic sources, are often bioactive and may cause adverse effects in aquatic biota.^1^ The environmental mixtures consist of thousands of chemicals, whereas their physicochemical properties, concentrations, and toxic potencies may range over several orders of magnitude.^2^ This challenge has been addressed by using bioanalytical approaches based on in vitro bioassays, which enable integration of the total biological activity of environmental mixtures.^3^ Despite the advances in LC- and GC-HRMS/MS techniques suited for wide-scope targeted screening of hundreds of compounds in a single analytical run,^4^ the effects of complex mixtures from environmental samples measured in vitro can often be only partly explained by the target chemicals.^5^ The unexplained fraction of the effect can be associated either with target compounds with unknown biological potency or with nontargeted chemicals.^6,7^ Many environmentally relevant contaminants still lack adequate data on their activity or potency in relation to key modes of action frequently detected in monitoring of aquatic environment, air, indoor dust, or biota samples.^8^

Interaction with the aryl hydrocarbon receptor (AhR) is a frequently observed molecular endpoint across various environmental matrices.^9,10^ This is of particular concern given the accumulating evidence linking AhR-mediated pathways to adverse effects on both ecosystem and human health.^11,12^ The AhR is a ligand-activated transcription factor that regulates the expression of genes involved in numerous physiological processes, including metabolism, immunity, organ development, embryogenesis, hematopoiesis, and neurodevelopment.^13,14^ It also plays a role in the pathogenesis of diseases, including autoimmune disorders,^15^ hepatic steatosis,^16^ cancer, and chemical toxicity, as it mediates the metabolism of small endogenous and exogenous molecules.^14^ AhR interacts with a wide variety of ligands of both natural and anthropogenic origin. Numerous environmental pollutants, including polycyclic aromatic hydrocarbons (PAHs), halogenated aromatic hydrocarbons, pesticides, and pharmaceuticals, interact with AhR as full agonists, partial agonists, or antagonists.^13,17^ AhR activation initiates the expression of detoxification enzymes that facilitate the oxidation and elimination of xenobiotics, helping to mitigate their potential toxic effects.^18^ Despite extensive research on the AhR and existing large data sets of AhR active compounds,^19^ numerous environmentally relevant and commonly occurring contaminants remain poorly characterized regarding their potential AhR-mediated activity (shortened to AhR activity in the following text). Since experimentally evaluating the potency of hundreds of compounds in each study is not feasible, modeling approaches can assist in bridging these major data gaps and identifying chemicals that contribute to the observed effects.^7,20^

Quantitative Structure–Activity Relationship (QSAR) is a modeling approach widely used in chemistry and toxicology to link molecular structures to various physicochemical properties and biological activities.^21–24^ Existing QSAR models for AhR activity were often developed for specific chemical groups like PCBs, PCDDs, and PCDFs, and thus have limited applicability, primarily focusing on predicting binding affinity.^25–27^ The limited diversity of these models restricts their use in predicting the effects of a broader range of environmental chemicals. Moreover, they do not distinguish between agonistic and antagonistic mechanisms of action, limiting their accuracy in predicting complete biological activity. QSAR models for AhR agonist activity, based on larger data sets, classify chemicals into two groups: active/inactive. These models primarily use large, quantitative high-throughput screening (qHTS) data sets from PubChem. For example, Goya-Jorge et al.^28^ created a balanced data set of 1837 compounds, with 804 active and 1033 inactive, while Klimenko et al.^19^ used three qHTS bioassays to compile a data set of 925 active and 204,513 inactive compounds. While predicting compounds as active or inactive is useful for initial screening, experimental characterization should be prioritized for those compounds with the highest predicted potency, as well as for those with lower predicted potency but that are present at high concentrations in environmental or human samples, given their potential role in the effects of environmental exposure mixtures.

In a recent study by Šauer et al.,^29^ water samples were collected using passive sampling along the Danube River as part of a long-term monitoring campaign. This approach captured a broad spectrum of compounds originating from diverse sources across the watershed that spans 10 countries and which is home to nearly 80 million inhabitants. The AhR agonist activity detected in the passive sampler extracts was linked to a comprehensive target chemical analysis, enabling the identification of potential contributors to the observed biological effects. Among the detected chemicals, 53 compounds with known AhR potencies accounted for, on average, less than 40% of the observed biological effect. In addition, 164 compounds with unknown AhR potencies from ToxCast were detected and are considered suspect contributors to the remaining activity. This gap in potency data highlights the need for QSAR predictions to improve the explanatory power of chemical analyses. Such predictions could help determine whether the detected target compounds sufficiently account for the observed AhR activity or whether significant contributions stem from chemicals outside the target list.

This study aimed to (i) develop a QSAR model for AhR activity levels using ToxCast data; (ii) prioritize water pollutants suspected of having AhR activity; (iii) validate the model with an external data set and experimental testing of the prioritized compounds; and (iv) apply the QSAR predictions to explain the observed effects of the environmental mixture based on the detected compounds. We aimed to develop a QSAR model capable of predicting multiple activity categories and to use the resulting values to address data gaps across extensive lists of target analytes in environmental samples. The model was applied to complex mixtures of micropollutants found in surface waters within a real-world environmental case study. This work integrates QSAR model development, experimental validation, and application to environmental data sets. We hypothesized that QSAR predictions of AhR potencies for a comprehensive list of measured target compounds in surface waters would enhance the explicability of the effects observed in the bioassays. A critical assessment of the approach was carried out to evaluate its strengths, limitations, and predictive value for complex environmental mixtures.

## Material and Methods

This study builds on our previous research,^29^ which involved a comprehensive chemical and effect-based assessment of water quality in the Danube River, with a focus on organic micropollutants and in vitro endocrine disruption endpoints. In this study, we develop and apply a novel QSAR model to predict the AhR potency of a wide spectrum of compounds detected across various sampling sites, with unavailable potency data. Compounds with the highest predicted AhR potencies were prioritized for experimental validation through in vitro bioassays. Both the QSAR predictions and experimental results were subsequently used to estimate the contribution of the detected target chemicals to the overall AhR-mediated activity observed in the environmental samples. An overview of the study workflow, including the key steps involved in the QSAR model development and application, is presented in Figure 1.

**Figure 1:**
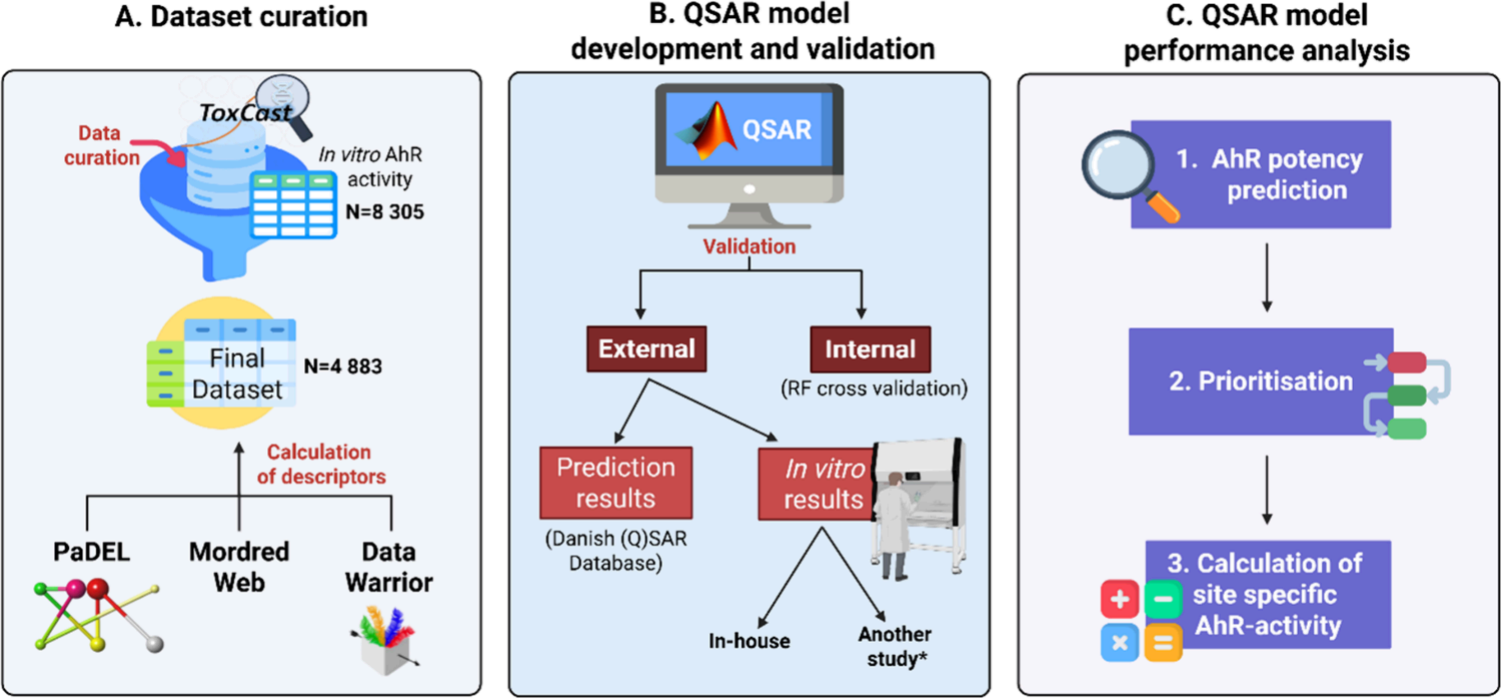
Workflow for the data set curation (A) and development-validation (B), and application (C) of the QSAR model to environmental monitoring data.

### Data Set Curation for the QSAR Model

The CompTox database by the US EPA was the source of experimental data for developing the QSAR model because it offers a consistent set of dose–response data from AhR agonism measurements generated by using a human-derived cell line comparable to our laboratory system. The data set is fully transparent and publicly accessible, providing all relevant metadata, data-analysis parameters, cytotoxicity data, and dose–response curves, which are particularly valuable for detailed quality control and verification. Data on the compound purity (Data set 1; tox21-ahr-p1) were retrieved from the NIH National Center for Advancing Translational Sciences website (https://tripod.nih.gov/pubdata/index.html, downloaded in August 2022). The data on AhR activity to build the QSAR model were retrieved from the CompTox database (https://comptox.epa.gov/dashboard/; version 2.1.1; downloaded in August 2022) with experimental data based on quantitative high-throughput screening of 8305 structures (10,496 data points). The following data sets were downloaded using an R package, ToxCast Analysis Pipeline (tcpl, version 2.0),^30^ reconstructing the data evaluation pipeline employed in the ToxCast data (SM1-Table S1 and Figure S1): TOX21_AhR_LUC_Agonist_viability with cytotoxicity (assay ID 807) as Data set 2; and TOX21_AhR_LUC_Agonist (assay ID 806) with AhRd activity as Data set 4. These assays exploit the human cancer liver cell line (HepG2) with a reporter gene responsive to AhR activation, followed by luminescence readout. Additional data set regarding cell viability/cytotoxicity was based on a study by Judson et al.^31^ and personal communication with the authors (Data set 3). These data are not specific to the AhR_LUC Agonist assay and were used to fill the gaps in Data set 2.

The data sets were curated by excluding data points with unreported or insufficient compound purity (<90%), as well as removing mixtures and inorganics. Effect concentrations (EC_20_ for cell viability and EC_25_ for AhR agonism) were calculated using curve-fitting models (Hill or Gain-Loss) in ToxCast. EC_20_ was used as a cytotoxicity limit, with compounds showing no significant effect on cell viability (<20%) deemed noncytotoxic. EC_25_ values were used to categorize compounds into four AhR activity groups (Inactive, Low, Medium, and High). AhR activity groups were defined by the distribution of the effective concentrations in the ToxCast data set to balance group size and coverage, with cut-offs reflecting data distribution rather than specific biological significance. Compounds were excluded if their cytotoxicity was within a factor of 3 compared to their EC_25_ for AhR activity (i.e., the EC_20_ for cytotoxicity was less than three times the EC_25_ for AhR activity), or if they were inactive on AhR but exhibited cytotoxicity with an EC_20_ below 100 μM. This filtering step ensured a data set of true positives and negatives not confounded by cytotoxic effects. Inactive compounds with undefined CAS registry numbers, compounds with EC_25_ between 100 and 1000 μM, and AhR-active compounds with inconsistent results across multiple experiments were also removed. The final data set was curated by manually checking dose–response curves, removing compounds with atypical curve shapes or low potency. The detailed steps of data curation are described in the Supporting Information (SM1-Section S1) and the final curated data set used for QSAR modeling is available in SM2-A1.

### Structural Predictors

EPAs Tox21 QSAR ready compound library in SDF format (ver. 2023-03-30_07_21_58) was used as a source of molecular structural data. DTXSID and ChID identifiers were used to connect two data sets, the toxicologically curated data set (SM1 Section S1) and QSAR-ready structural data. This merged data set was recurated from the perspective of molecular structures for QSAR modeling. Solvents, nonorganic fragments, extraneous molecular pieces, and duplicated molecules were removed from entries using Open Babel 3.1.1, and UMSA Galaxy Chemical toolbox, and the final database was additionally inspected using OSIRIS DataWarrior 5.5.0.^32^ Mordred molecular descriptor calculator v1.2.0 ^33^ was used for processing the database, i.e., 3D structures of molecules were generated, and salts were removed. Compounds, where descriptors were not generated (i.e., inputs for molecules were not accepted for some reason), were manually checked. PaDEL-Descriptor 2.21 ^34^ was used for generating 496 molecular PubChem structural keys for chemicals. A total of 1825 molecular descriptors were generated using Mordred from the structural data file for these chemicals as well. Molecular descriptors and fingerprints with missing values or variability of less than 1% were excluded. After data curation, a total of 777 predictors were used for the model, including 297 molecular descriptors (from Mordred and DataWarrior) and 496 PubChem structural keys.

### QSAR Modeling Approach

QSAR modeling was based on the nonparametric Random Forest (RF) method.^35^ RF is commonly used for QSAR modeling because it is suitable for large sets of predictors of various types, which is advantageous when combining fingerprints and molecular descriptors for prediction. One of the main advantages of RF is its ability to handle correlated predictors without the need for multicollinearity-based predictor selection before running the model.^36^ Compared with tree-based boosting algorithms (e.g., XGBoost or LightGBM), RF is generally less sensitive to hyperparameter tuning, less prone to overfitting on small or imbalanced QSAR data sets, and provides interpretable feature importance measures. The principle of the method is based on the creation of a random forest, which consists of a set of a large number of CART-type binary decision trees,^37^ where the data set is divided into a test set and a training set. The training set for each tree is created by bootstrap selection with repetition. Observations that were not selected by the bootstrap selection (about 1/3 of the data set) are used as test sets for error estimation (oob, out-of-bag, out of bootstrap sample estimates). Thus, when forests are used for classification, each tree provides information on how each observation is classified into the resulting category. The outcome of the forest classification is determined by the majority vote of all trees. The random forest was tested for different parameter settings: category weights, the number of samples in the terminal node of the tree, the number of randomly selected predictors, and the number of trees. The selection of optimal parameters was performed using Bayesian optimization.^38^ The model was initially run using all predictors (molecular descriptors and fingerprints), and their importance was evaluated based on the misclassification error. Subsequently, the model was run again only with predictors whose importance was at least 0.05 relative to the most significant variable, with a value of 1. The AhR QSAR model was evaluated using overall accuracy (OA), weighted overall accuracy (WOA), sensitivity, specificity, precision, and Matthews correlation coefficient (MCC) for both the training and testing sets. In addition, the probability of classifying each compound into each activity category (class probability; ranging from 0 to 1) was determined using a probability matrix calculated as the proportion of observations assigned to each category. The class probability, together with sensitivity and specificity measurements were used to prioritize the compounds and define the applicability domain. The class probability from the random forest was shown to be an appropriate metric for the applicability domain.^39^

Since the data set is highly imbalanced, with the number of active compounds approximately ten times lower than that of inactive compounds, various weight settings were tested for random sampling in the model. The weights of individual AhR activity categories and other model hyperparameters were tested using a combination of a systematic grid search with defined step sizes and Bayesian optimization. The optimal category weights were determined based on the model configuration with the highest WOA after parameter tuning. The procedure and full parameter settings used for tuning are provided in the Supporting Information (SM1-Section 6 and Table S3).

Several approaches were used for validation: (i) internal, the random forest provides internal validation using OOB (out-of-bag) samples obtained through bootstrap selection to provide an unbiased estimate of the test set error, and (ii) external, using results from our in vitro bioassay testing and comparison with an independent experimental data set from Goya-Jorge et al.^28^ For validation with the external data set from Goya-Jorge et al.,^28^ only a binary comparison of active and inactive compounds could be performed. The model was created and tested in MATLAB R2022a.

### Data Set for Prioritization and Linking of AhR Activity to Target Chemicals

The newly developed QSAR model was applied to predict the AhR activity of 164 compounds with previously unknown potency, identified in the Joint Danube Survey 4 (JDS4), a comprehensive monitoring campaign along the Danube River,^29^ to prioritize the most relevant compounds for experimental validation in an in vitro bioassay (SM1-Section S2.1, SM1-Figures S2, and S3). In the study by Šauer et al.,^29^ surface water extracts representing a longer-term pollution situation were collected using 2 types of passive samplers, i.e., HLB (Hydrophilic Lipophilic-Balanced sorbent) for polar compounds and SR (Silicone rubber) for nonpolar compounds. Their extracts were analyzed for various contaminants, including pesticides, pharmaceuticals, PAHs, PCBs, and PBDEs. AhR activity of extracts was assessed in an in vitro bioassay (SM-Section S3) and the mixture modeling approach based on bioanalytical equivalent concentrations (BEQs) was applied to determine the contribution of detected compounds to the observed effects, using the relative effect potency (REP) for each compound derived from the ToxCast data (SM-Section S2.1). Of the 456 detected compounds, 53 active chemicals contributed to the observed AhR effect, accounting for an average of 23 and 37% of the bioactivity in different sampler types. There was no available information about potential AhR activity for 164 detected compounds. A detailed description of the Danube data set is provided in SM1-Section S2.1. After the QSAR model was applied, the contribution of all detected compounds to the observed AhR-mediated activity at individual sampling sites was calculated again using their detected concentration and QSAR-predicted AhR activity and EC_25_ values determined from the experimental testing of the prioritized compounds (SM1-Section S2.2).

### In Vitro Bioassay to Assess AhR Potency

AhR-mediated activity of prioritized compounds with predicted AhR potency was assessed using the same Caflux in vitro model derived from rat hepatoma stably transfected with AhR-responsive green fluorescent protein (GFP) reporter (H1L1.1c2)^40^ as was used for the detection of this bioactivity in the samples from the Joint Danube Survey (described in Šauer et al.^29^ and in SM1-Section S3).

## Results

### Data Set Curation for the QSAR Model

The data curation pipeline excluded 3 022 (38%) of the compounds. Details on the data curation are available in SM1-Section S4 and Table S2. The curated set (SM2-A1) contained 4 883 compounds that were divided according to their AhR activity into four categories: inactive, low activity (EC_25_ = 11–100 μM), medium activity (EC_25_ = 2.04–10 μM), and high activity (EC_25_ = 0.0001–2.03 μM). The representation of AhR-active and -inactive compounds in the ToxCast database is highly imbalanced (Table 1). The number of inactive compounds is about 10-fold higher than that of AhR active compounds. No acyclic (with a chain and without a ring) or cyclic compounds (with a nonaromatic ring) belonged to the category with the highest AhR activity. Only 1 acyclic and 3 cyclic compounds were represented in the group of compounds with Medium AhR activity. In the Low activity group, 7 acyclic and 12 cyclic compounds were identified (Table 1). Highly AhR active molecules from the ToxCast database contain mostly 3–4 or more aromatic rings (SM1-Figure S4).

**Table 1:** Compound Counts in the ToxCast Dataset Related to AhR Activity, Indicating Inclusion or Exclusion in the QSAR Models, Distribution across 2-, 3-, and 4-Category AhR Activity Classes, and the Presence and Types of Ring Structures

		excluded^a^	included in model			
*N* AhR	activity categories	total	total	without ring	nonaromatic ring	aromatic ring
2	inactive (>1000)	2499	4457	982 (22%)	838 (19%)	2637 (59%)
	active (<100)	523	426	8 (1.9%)	15 (3.5%)	403 (95%)
3	inactive (>1000)	2499	4457	982 (22%)	838 (19%)	2637 (59%)
	low (11–100)	228	276	7 (2.5%)	12 (4.3%)	257 (93%)
	medium-high (<10)	98	150	1 (0.6%)	3 (2%)	146 (97%)
4	inactive (>1000)	2499	4 457	982 (22%)	838 (19%)	2637 (59%)
	low (11–100)	228	276	7 (2.6%)	12 (4.4%)	257 (93%)
	medium (2.04–10)	61	103	1 (1%)	3 (2.9%)	99 (96%)
	high (<2.04)	37	47	0 (0%)	0 (0%)	47 (100%)
	total *N*	3022	4 883	990 (20%)	853 (18%)	3040 (62%)

a Excluded compounds due to insufficient compound purity, cytotoxicity or EC_25_ in range 100–1000 μM; activity categories in the models according to EC25 (μM); *N* AhR—number of AhR categories in model.

Based on this distribution of AhR active compounds, the presence of an aromatic ring was further used as a structural feature for reducing the database size for building the QSAR model, i.e., compounds from the curated data set were grouped based on the presence of a ring in their molecular structure (Table 1).

### Model Results

The initial data set for model building included all compounds, but since the active compounds were predominantly aromatic, additional models were created specifically for aromatic compounds to better balance the representation of active and inactive compounds. A total of five different QSAR models were trained; three models for aromatic compounds, where it was possible to divide the compounds into up to four levels of AhR activity, namely (i) M_arom 2—two categories (Inactive/Active), (ii) M_arom 3—three categories (Inactive, Low and Medium + High activity), and M_arom 4 (iii)—four categories (Inactive, Low, Medium, and High activity), and two models for all compounds, where the division was into a maximum of three levels of activity due to the greater prevalence of inactive compounds, namely (iv) M_ALL 2—two categories (Inactive/Active) and (v) M_ALL 3—three categories (Inactive, Low and Medium + High activity) (Table 1). In models with three AhR activity categories (M_ALL 3 and M_arom 3), compounds from the Medium and High categories were merged. Model hyperparameter settings are detailed in the Supporting Information (SM1-Table S3).

For comparison, goodness-of-fit parameters were calculated for models with three and four categories of AhR activity alongside the two-category models, where active compounds in the categories of Low, Medium, and High activity were grouped together as active. All five models showed high sensitivity (correctly classified active compounds) and specificity (correctly classified inactive compounds), with a weighted overall accuracy (WOA) ranging from 77 to 86.9%. However, the models had a relatively low precision. Table 2 presents the results of the AhR activity classification models on the test sets, with details for the different AhR activity categories shown in SM1-Table S4.

**Table 2:** Performance of Classification Models on Test Sets Based on All (ALL) or Solely Aromatic Compounds (Arom)^a^ ^b^

model	OA %	WOA %	sensitivity %	precision %	specificity %	NPV %	MCC
M_ALL 2	84.2	86.9	90.1	34.5	83.7	98.9	0.5
M_ALL 3	79.2	82.0	83.6	29.0	80.4	98.1	0.42
M_arom 2	76.1	77.0	78.2	33.1	75.8	95.8	0.39
M_arom 3	85.4	78.1	66.3	50.2	90.0	94.6	0.5
M_arom 4	81.9	78.4	66.1	46.0	87.6	94.9	0.46

a All parameters are calculated only for the main division into active and inactive compounds.

b TA (true active), TI (true inactive), FA (false active), FI (false inactive), Overall accuracy (OA) = (TA + TI)/(TA + TI + FI + FA), Weighted overall accuracy (WOA) = (sensitivity + specificity)/2, Sensitivity (Recall) = TA/(TA + FI), Specificity = TI/(TI + FA), Precision = TA/(TA + FA), Negative predictive value (NPV) = TI/(TI + FI), Matthews correlation coefficient $\mathrm{MCC}=\frac{\left(\mathrm{TA}\times \mathrm{TI}-\mathrm{FA}\times \mathrm{FI}\right)}{\sqrt{\left(\mathrm{TA}+\mathrm{FA}\right)\left(\mathrm{TA}+\mathrm{FI}\right)\left(\mathrm{TI}+\mathrm{FA}\right)\left(\mathrm{TI}+\mathrm{FI}\right)}}$ . M_ALL 2—model built with all compounds in 2 activity categories, M_ALL 3—model built with all compounds in 3 activity categories, M_arom 2—model built with aromatic compounds in 2 activity categories, M_arom 3—model built with aromatic compounds in 3 activity categories, M_arom 4—model built with aromatic compounds in 4 activity categories, active compounds in the categories of low, medium, and high activity were grouped together as active.

For the two-category AhR activity model (M_ALL 2), the model classified 90.1% of active compounds correctly but with low precision (34.5%). The three-category models (M_ALL 3 and M_arom 3) had comparable overall and weighted overall accuracies, but the model for aromatic compounds (M_arom 3) showed a higher precision (50.2%). The sensitivity for the medium-high category was 89.7% in M_arom 3 and 100% in M_ALL 3. In contrast, compounds with the low AhR activity showed the lowest sensitivity (36.6% in M_arom 3 and 47.5% in M_ALL 3), with considerable overlap with inactive compounds (SM1-Table S4).

The model M_arom 4, with four categories of AhR activity, achieved overall accuracies of 81.9% and weighted accuracies of 62.8% (SM1-Table S4). The model correctly classified all compounds with the highest AhR activity (100% accuracy) with a precision of 38.5% and 12.3% false active compounds; no high activity compounds were classified as inactive. Classification accuracy for compounds with Medium AhR activity was low (19.2%), and they were largely classified in the High activity category, which may lead to an overestimation of the AhR activity level. However, only 13.1% of the Medium AhR active compounds were classified as inactive, indicating that the model accurately classified these compounds as active at other activity levels. Compounds with Low activity were correctly classified 44.4% of the time, with a significant portion classified as inactive. A larger percentage of false active compounds (48.9–68%) was observed in the Medium and Low activity categories.

Although the four-category model struggles with the classification of compounds with Medium AhR activity, at the same time, splitting the categories into Medium and High improves the accuracy and precision for the compounds with the highest AhR activity. Detailed results of classification into three and four categories of AhR activity on the test set are shown in SM1-Table S4. The MCC values (0.39–0.50) indicate overall moderate classification reliability for all evaluated models, which corresponds to their high accuracy but also relatively low precision. The M_ALL 2, M_arom 3, and M_arom 4 models achieved the highest MCC values (0.46–0.50) and provided the most balanced performance despite a higher rate of false positives in specific AhR activity categories.

For final classification and prioritization, we used the model for three AhR activity categories (M_ALL 3) for all compounds and the model for four categories (M_arom 4) for aromatic compounds. The first model is suitable for predicting compounds without an aromatic ring, while the second model helps to increase precision (reduce false positives) for aromatic compounds with the highest activity.

### Prioritization of Compounds

The QSAR model assigned each compound with a predicted AhR activity a priority for experimental testing. Prioritization combines both the AhR activity level of the compounds and the accuracy of the model. The priority ranking was determined by calculating the probability of assigning each compound to each AhR activity category. Compounds were classified into AhR activity categories based on the highest probability. Truly active compounds classified to a given category with high probability are also better defined based on structural predictors. Based on the predicted activity and the calculated probability of classification, the compounds were prioritized into four priority levels. The prioritization procedure for the evaluation of compounds for the three and four activity categories is presented in Table 3. Compounds with priority 4 are the most potent AhR agonists that are predicted by the model with high sensitivity and precision. Compounds with priority 3 are Medium/Medium-High/High AhR active compounds that are predicted by the model with a little lower precision. Priority group 2 includes Low AhR active compounds classified with high sensitivity and precision but also compounds with Medium/Medium-High AhR activity, which have lower sensitivity and precision. Priority group 1 includes Low AhR active compounds classified with high sensitivity but lower precision. Inactive compounds are assigned to priority group 0. Group “not reliable” (NR) contains compounds with low accuracy and sensitivity of AhR activity prediction; these compounds may be out of the domain of the chemical space of the training data or cannot be distinguished by structural predictors.

**Table 3:** Procedure for Prioritizing Compounds

priority	probability of classification of the compound to a given AhR activity category	model
4	compounds classified in the High AhR activity category with probability ≥ 0.5	M_arom 4
	compounds classified in the Medium/Medium-High AhR activity category with probability ≥ 0.5 and probability <0.1 for inactive category	M_arom 4
		M_ALL 3
3	compounds classified in the Medium/Medium-High/High AhR activity category with probability ≥ 0.4 and probability <0.1 for inactive category	M_arom 4
		M_ALL 3
2	compounds classified in the Low AhR activity probability with probability ≥ 0.6 and probability <0.1 for inactive category	M_arom 4
	compounds classified in the Medium/Medium-High AhR activity category with probability ≥ 0.4 and probability <0.1 for inactive category	M_ALL 3
1	compounds classified in the Low AhR activity category with probability ≥ 0.7	M_arom 4
		M_ALL 3
0	compounds classified as Inactive with probability ≥ 0.6	M_arom 4
		M_ALL 3
NR (not reliable)	if the model result does not meet the above criteria	M_arom 4
		M_ALL_3

### In Vitro Testing of the Prioritized Compounds

A prioritization scheme was applied to select candidate compounds for bioassay testing from a total of 164 compounds detected in the Danube River with concentrations above the detection limit. Out of 164 compounds, 14 were predicted by our model to be AhR active (Low = 4, Medium = 2, Medium-High = 2, High = 6), 106 were inactive, and for 44 compounds, the model results were unreliable (SM2-A2).

In total, we selected 18 compounds for laboratory testing of the predicted activity. This list included all compounds with predicted highest activity (Medium, High) and priority (4 and 3), and several compounds with Low activity or Inactive. The compounds from the Low and Inactive categories were selected on the basis of direct availability of their standards in sufficient quantity and purity for bioactivity testing in our laboratory. For cyclopenta­[cd]­pyrene (from the High activity category and priority 4), we had to use the experimental value from the literature, since the standard for biotesting was not available. The results of AhR activity prediction were compared with the Caflux biotest results (Table 4). The best agreement was observed for compounds with a high AhR activity. Among the six compounds with High predicted activity, the biotest confirmed High activity in four cases, while one compound exhibited Medium activity and one compound exhibited Low activity. Of the four compounds with predicted Medium (Medium-High) activity, one compound elicited Medium activity, one compound Low, and two compounds were cytotoxic. In the predicted Inactive category with 7 tested compounds, only one could be confirmed as clearly inactive up to the greatest tested concentration of 500 μM, whereas six were inactive but also cytotoxic within the tested range. High incidence of cytotoxicity hindered the confirmation of the AhR activity prediction, and it was identified as a potential confounding factor (detailed discussion SM1-Section S9).

**Table 4:** Results of In Vitro Testing (EC_25_ in μM and REP_EC25_) of the Prioritized Compounds and Comparison with the QSAR Model Prediction^a^

		in vitro testing			QSAR model	
		mean ± SD				
CAS	chemical name	EC_25_ (μM)	REP_EC25_ ^d^	AhR activity	AhR activity	priority
205-82-3	benzo[*j*]fluoranthene	2.43 × 10^–03^ ± 4.57 × 10^–04^	8.39 × 10^–04^ ± 2.36 × 10^–04^	High	High	4
193-39-5	indeno[123-*cd*]pyrene	9.30 × 10^–03^ ± 2.55 × 10^–03^	2.19 × 10^–04^ ± 3.97 × 10^–05^	High	High	4
243-17-4	benzo[*b*]fluorene	0.23 ± 0.08	9.43 × 10^–06^ ± 4.34 × 10^–06^	High	High	4
239-35-0	benzo[*n*]aphthothiophene	0.40 ± 0.23	6.81 × 10^–06^ ± 1.15 × 10^–06^	High	High	4
578-95-0	acridone^b^	6.84 ± 2.30 × 10^–03^	4.75 × 10^–07^ ± 1.64 × 10^–08^	Medium	Medium-High	3
198-55-0	perylene	19.1 ± 13.8	1.37 × 10^–07^ ± 9.70 × 10^–08^	Low	High	4
27208-37-3	cyclopenta[*cd*]pyrene^c^		6.53 × 10^–07^	Medium	High	4
217-59-4	triphenylene	32.5 ± 12.1	8.64 × 10^–08^ ± 4.10 × 10^–08^	Low	Medium	3
105650-23-5	2-amino-1-methyl-6-phenylimidazo[4,5-*b*]pyridine (PhIP)	>0.70		Cytotoxic	Medium-High	3
442-51-3	harmine	>0.64		Cytotoxic	Medium	3
486-84-0	harman	>1.25		Cytotoxic	Low	3
59729-33-8	citalopram	>50		Cytotoxic	Inactive	0
18323-44-9	clindamycin	>500		Inactive	Inactive	0
116539-59-4	duloxetine	>4		Cytotoxic	Inactive	0
67129-08-2	metazachlor	>10		Cytotoxic	Inactive	0
525-66-6	propranolol	>62.5		Cytotoxic	Inactive	0
79617-96-2	sertraline	>1		Cytotoxic	Inactive	0
106700-29-2	pethoxamid	>50		Cytotoxic	Inactive	0

a EC_25_ (effective concentration that induces 25% of the maximum fluorescence elicited by the calibration curve of the reference substance-TCDD); REP_EC25_ (relative effect potency of the substance at EC_25_); AhR activity category (EC_25_ in μM): Inactive (>1000), Low (10–100), Medium (2.04–10), High (0.0001–2.03), Medium-High (0.0001–10), NR (not reliable); SD (standard deviation).

b EC_25_ was extrapolated from a curve fitted to data where mean maximal induction reached 10%.

c Cyclopenta­[*cd*]­pyrene was not tested, experimental results were taken from a study by Machala et al.

d REP_EC25_ was determined as a ratio of EC_25_ of reference AhR agonist TCDD and EC_25_ of a compound resulting from the experimental measurement.

The biotesting results confirmed the model’s ability to accurately identify active compounds and predict their activity levels. Nonetheless, additional testing with a broader set of compounds is required for comprehensive validation.

### External Validation and Comparison of Prediction Results with the Danish (Q)­SAR Database and Literature

Our QSAR model was validated using an external data set from Goya-Jorge et al.^28^ Due to the absence of EC_25_ values in their study and the use of a different reference compound, a direct comparison with our results was only feasible using binary classification (active/inactive). In their work, compounds were considered active if they induced AhR activation greater than 10% (relative to the positive control—FICZ: 5,11-dihydro-indolo­[3,2-*b*]­carbazole-6-carboxaldehyde) within the tested concentration range of 5–100 μM. To compare our predictions with the results of 40 in vitro-tested compounds from their study, our models (M_ALL3/M_arom 4) provided reliable predictions for 30 compounds, while predictions for the remaining 10 were considered unreliable. Among the 30 reliable predictions, M_ALL 3/M_arom 4 showed a 90% agreement with in vitro-tested compounds from Goya-Jorge et al.^28^ Specifically, of 11 in vitro tested as active compounds, 10 were correctly predicted as active by our model, with one misclassified as inactive. Of the 18 compounds in vitro-tested as inactive, 16 were correctly predicted as inactive, while 2 were misclassified as active. The comparison with the external data set and detailed validation results is provided in SM2-A4.

The Danish (Q)­SAR database (http://qsar.food.dtu.dk) provided alternative AhR activity predictions for 164 compounds detected in the Danube, which were compared with our model outputs (SM1-Section S7.1 and SM2-A2). Two categories of AhR active and inactive compounds were used for comparison. AhR activity prediction was available for 58 and 66 compounds in the case of the random and rational models in the Danish (Q)­SAR database, respectively; 45 compounds were not found in the Danish (Q)­SAR database, and another 53 compounds (random model) and 61 compounds (rational model) were out of the applicability domain (SM1-Table S5). All compounds that were predicted as AhR inactive by the Danish QSAR model were also inactive in our QSAR model. Only four compounds were predicted as active by the Danish model (random model), and these compounds were also predicted as active by our model. The other compounds predicted as active by our model were out of domain in the Danish model, and one was not found in the Danish database at all. For compounds with available predictions from both models, the Danish model (random) and our model showed complete agreement (100%). Our model was able to predict 73.2% of the compounds detected in the water samples from a major European river, compared to the Danish models, where predictions were available only for 35.4 and 40.2% of them, respectively. The difference in the coverage of the predictions is due to the difference in the compounds used to train the Danish (PubChem database) and our model (ToxCast database). Although the training data sets for the Danish QSAR model (*N* = 4625) and for our QSAR model (*N* = 4883) are similar in size, there is almost no overlap between them. The data sets share only 18 compounds, of which 13 are inactive, and 5 are active (1 Low, 2 Middle, and 2 High) (SM1-Figure S6). This suggests that the two models are complementary, each potentially predicting the activity for different types of structurally distinct compounds.

Open scientific literature was searched to gather additional evidence on AhR activity for the 164 compounds detected in the Danube River that were not covered by the ToxCast database. Information was collected broadly, encompassing various biological models and measured endpoints. While these additional data indicate potential interaction or noninteraction with the AhR receptor, they are not directly comparable to our experimental or predicted results and are therefore treated as supplementary evidence. Literature data on AhR activity were available for 64 (39%) compounds (*N* = 164), of which 42 compounds had a reliable prediction from our model. The literature data agreed with our prediction (M_ALL 3 and M_arom 4 for aromatic compounds) in the case of 28 compounds (67%). A detailed comparison with the Danish QSAR model and the literature is provided in SM1-Section S7.2 and SM2-A2.

### Linking of AhR Activity to Target Chemicals with QSAR Model Predictions and Experimental Data

The QSAR model predictions and new experimental data were used to refine the calculation of compounds’ contribution to AhR-activity at Danube River sites using the BEQ_chem_ (Bioanalytical equivalent based on chemical analyses) approach. The predicted REP (Relative effect potency) values for each activity category were determined using the geometric mean of EC_25_ values of compounds in the particular category in the Toxcast data set (i.e., EC_25_ of 32 μM for Low, 5 μM for Medium, 2.5 μM for Medium-High, and 0.5 μM for High). Two scenarios were used to calculate the contribution of detected compounds to site-specific AhR activity: scenario 1 with QSAR model predictions only and scenario 2 with QSAR model predictions and experimentally determined REPs for the prioritized compounds tested in vitro. Detailed description of the calculation methodology and model scenarios can be found in the Supporting Information (SM1-Sections S2.2 and S8). Complete results of calculations using both scenarios can be found in SM2-A3.

Before applying the QSAR model, a total of 53 compounds (of 456) detected contributed to the observed activity, ranging from 26–36 contributing compounds per site in Hydrophilic Lipophilic-Balanced sorbent (HLB) and 10 contributing compounds per site in silicone rubber (SR) samplers). These compounds were responsible for 7–108 and 14–78% of the total observed AhR activity calculated in the Danube study, for the HLB and SR samplers, respectively.^29^ The effect explicability was markedly higher at Site 5 Panevo, which can be considered an outlier due to high levels of insecticide diflubenzuron, food additive piperine, and benzo­[*k*]­fluoranthene (PAHs), as this site had a higher contamination burden in general. With the QSAR model, an additional 4 to 8 AhR-active compounds were identified, depending on the scenario. This increased total explicability of AhR activity by up to 10.5% for the HLB sampler (Figure 2) and 7.9% for the SR sampler (Figure 3), with the increase varying by site.

**Figure 2:**
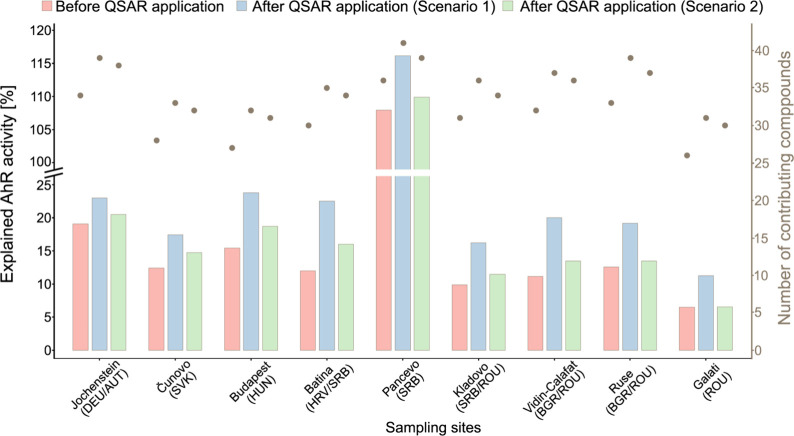
Proportion of observed AhR activity [%] explained by compounds detected in hydrophilic lipophilic-balanced sorbent (HLB) samplers, before and after application of QSAR model predictions, under two scenarios (left *y*-axis; note axis break between 25 and 100%). In Scenario 1, AhR activity was derived from QSAR model predictions; in Scenario 2, experimental results for tested compounds were used in place of model predictions. Gray points represent the number of detected compounds contributing to the AhR activity in each scenario (right *y*-axis).

**Figure 3:**
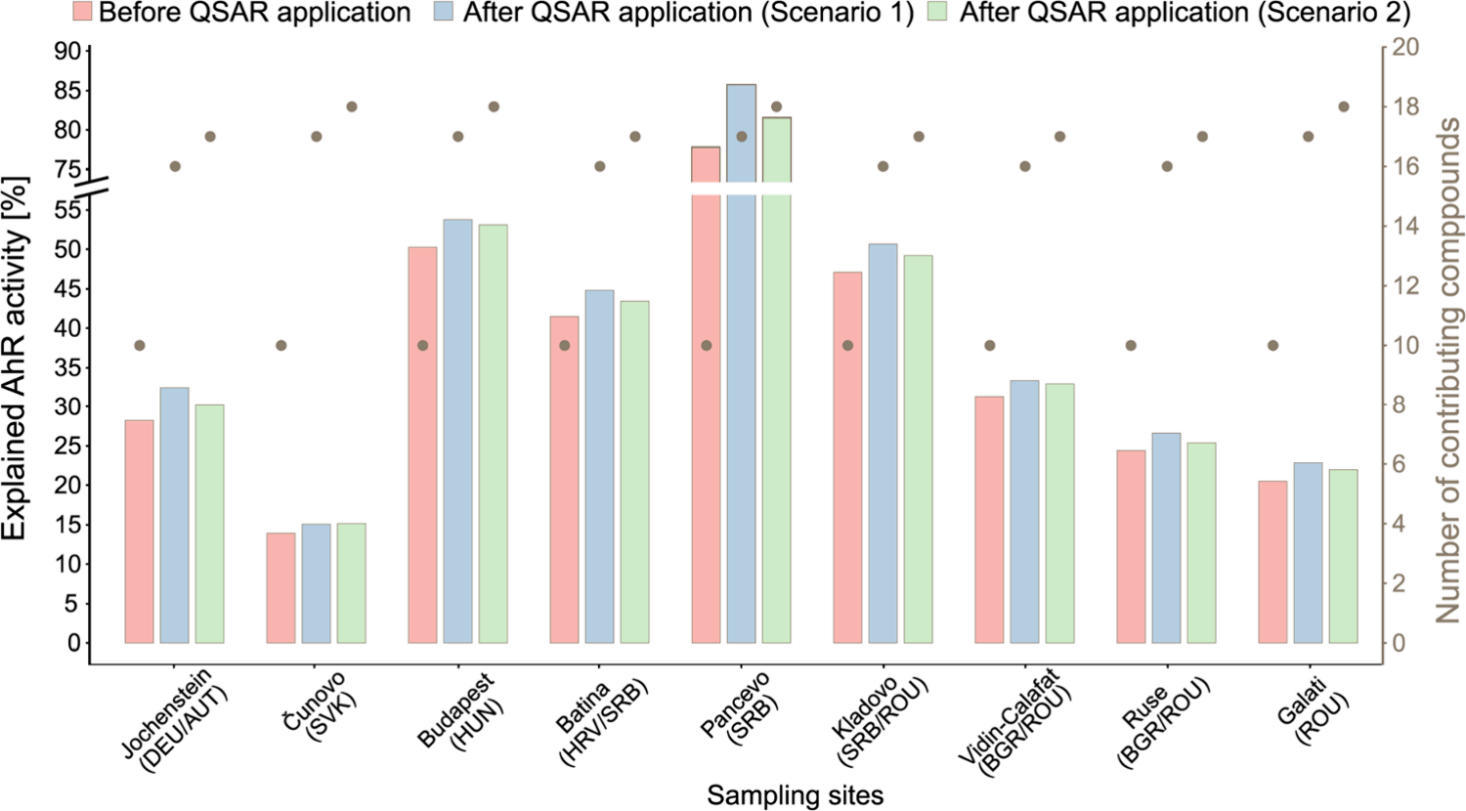
Proportion of observed AhR activity [%] explained by compounds detected in Silicone Rubber (SR) samplers, before and after application of QSAR model predictions, under two scenarios (left *y*-axis; note axis break between 55% and 75%). In Scenario 1, AhR activity was derived from QSAR model predictions; in Scenario 2, experimental results for tested compounds were used in place of model predictions. Gray points represent the number of detected compounds contributing to the AhR activity in each scenario (right *y*-axis).

However, a total of 44 compounds could not be included in the calculation of BEQ_chem_, as their classification of the compounds by the QSAR model was unreliable. A larger average increase to the explained portion of the observed AhR activity with model prediction (Scenario 1) was observed in the case of HLB sampler (7%, SM2-A3) and smaller with SR sampler (3.6%, SM2-A3).

In the case of Scenario 2, the increase of the explained AhR activity reached only ∼2% (for both samplers) due to the exclusion of cytotoxic compounds (PhIP, harmine, and harmane, Table 4) using the same logic as in the case of the ToxCast data. However, given the relatively high concentrations of these compounds at certain sites, their true contribution to the observed AhR activity may be underestimated. Cytotoxicity interfered with experimental validation for nine compounds, limiting the ability to confirm predicted AhR activities and thereby adding uncertainty to the overall result of scenario 2 based on experimental data (Figures 2 and 3).

## Discussion

### Data Curation for Reliable QSAR Modeling

Through extensive data curation and the application of strict criteria for data inclusion in the QSAR model, we generated a reliable data set of effect data, which is crucial for ensuring the quality of QSAR models.^41^ The first step was the curation of the structural descriptors for the compounds in the model. Previous studies have shown that accurate structure representation has a far greater impact on QSAR model prediction performance than the choice of optimization techniques.^42^ In fact, even minor errors in chemical structures can significantly reduce the accuracy of model predictions.^43^ The curation of effect data involved several steps that led to the exclusion of compounds burdened by uncertainty due to issues such as unknown and insufficient purity, cytotoxicity, or experimental error, factors that have been shown to be crucial for improving the prediction quality of QSAR models.^44,45^ In this study, 11% of compounds were excluded from the experimental data set due to cytotoxicity, which was shown to be the most prominent confounding factor in the high throughput screening of receptor binding. This may result in a significant portion of false positives especially in the antagonist mode and false negatives in agonistic mode.^46^ In our data curation scheme, we applied a strict exclusion criterion for cytotoxic compounds (EC_25_ for AhR activity at least 3 times lower than EC_20_ for cytotoxicity) to reflect the phenomenon of cytotoxicity burst and eliminate false positives. Cytotoxicity burst refers to the nonspecific activation of the reporter gene in cells close to their death, which is associated with triggering cell stress pathways, chemical reactivity, physicochemical disruption of proteins or membranes, or broad low-affinity noncovalent interactions.^31,47^ During the experimental testing of prioritized compounds, we frequently encountered cytotoxicity, which hindered our ability to confirm their predicted activity or inactivity. This issue was particularly problematic when cytotoxic effects occurred at low concentrations. Another hindrance of the experimental testing and activity confirmation may be low solubility of some compounds in aqueous media.^47^ QSAR predictions may fall above the solubility limit since the model does not reflect compound solubility. Integrating QSAR models with solubility predictions or experimental solubility data can enhance the result accuracy and reliability. Especially in the case of inactive compounds, experimentally confirming the absence of activity up to 1 000 μM proved challenging for many frequently occurring environmental micropollutants due to the limitations mentioned above.

To achieve robust results, a combination of available assays for a given biological end point is typically used^48,49^ but this becomes challenging when determining activity categories based on effective concentration. In our study, the activity groups were determined solely by the distribution of values within the data set rather than by any underlying biological rationale, which also makes such thresholds difficult to transfer across different assays. Additionally, for measurements from the ToxCast database, if replicate assay measurements were available, then a methodology was applied to exclude inconsistent results. However, for most compounds, only a single measurement without replicates was available (80.3%), which could be a source of uncertainty in the training data set.

### Structural Features of AhR Agonist Activity

Clearly defining the molecular structures or features associated with AhR activity is challenging due to the high structural diversity of ligands, the receptor’s promiscuity, and the likely existence of at least two distinct binding modes.^50^ While AhR activity is generally associated with hydrophobic compounds, several studies have shown that midpolar and polar AhR ligands can also play a significant role in environmental matrices.^29,51–53^ This is also represented by the high octanol–water partition coefficient increasing from AhR inactive to highly active compounds in the ToxCast database (SM1-Figure S4). The AhR activity is sometimes referred to as dioxin-like activity according to the historically well-known potent ligand TCDD.^54^ Many AhR agonists are planar molecules, e.g., PAHs and dioxin-like compounds (DLCs), such as PCBs, PCDDs, and PCDFs.^55^ The presence of rotatable bonds is a surrogate for the flexibility of molecules, which has a substantial influence on the affinity and specificity when binding to a protein. In our data set, highly active molecules contain mostly only 2–3 rotatable bonds (SM1-Figure S4). In cases where molecules contain aromatic ring systems, planarity can typically be achieved only when the number of rotatable bonds is low, e.g., in PCBs with few rotatable bonds or in fully rigid molecules with zero rotatable bonds, such as TCDD. The presence of an aromatic ring and an electron acceptor group plays a role in AhR activity of both agonists and antagonists.^28^ There is a high structural diversity of AhR ligands with the presence of heteroarenes, halogenated biphenyls, or polycyclic aromatic hydrocarbons (and their derivatives) where angular benzo-rings form a bay-region.^56,57^ Highly active molecules from the ToxCast database contain mostly 3–4 aromatic rings or more (SM1-Figure S4). Therefore, the presence of an aromatic ring is one of the most important descriptors, which can be used for balancing databases where inactive compounds (mostly aliphatic structures) significantly exceed AhR active compounds, as in our case (Table 1). The structural diversity of AhR activators is also reflected by the 3D-MoRSE (3D-Molecule Representation of Structures based on Electron diffraction) descriptors. 3D-MoRSE molecular descriptors represent the spatial and electronic properties of molecules,^58^ whose values varied between levels of AhR activity in our data set as well and were found as important predictors in our model (SM1-Section S5 and Figure S4).

### Model Performance and Validation

A crucial aspect of regulatory acceptance of alternative methods involves having clear requirements for their validation, as well as identifying the type of information necessary for their implementation. In November 2004, the 37th OECD’s Joint Meeting of the Chemicals Committee and the Working Party on Chemicals, Pesticides and Biotechnology agreed on the OECD Principles for the Validation of (Q)­SAR models for regulatory purposes. The agreed OECD Principles state that “to facilitate the consideration of a (Q)­SAR model for regulatory purposes, it should be associated with the following information: a defined end point; an unambiguous algorithm; a defined domain of applicability; appropriate measures of goodness-of-fit, robustness, and predictivity; a mechanistic interpretation, if possible. In developing our QSAR model, we aimed to meet these requirements, which are fundamental for regulatory assessment of QSAR models in the (Q)­SAR Assessment Framework,^59^ and follow best practices.^60^ Where this was not fully achievable, the challenges are discussed below.

From the point of view of using the model for the prioritization of compounds, an important indicator is Sensitivity, which basically indicates the ability of the model to capture active compounds, but an equally important indicator is Precision, which indicates the proportion of predicted active compounds that were truly active. The issue of a high percentage of false positives in our model is particularly evident in the prioritization of compounds for testing, where many predicted active compounds may actually be inactive. This high false positive rate arises from the large number of AhR-inactive compounds in the ToxCast database, which are structurally similar to the AhR-active compounds. By excluding some structurally similar inactive compounds to reduce the unbalanced modeling ensemble (i.e., the ratio of active to nonactive compounds), which is a common practice in QSAR models, false positives could be artificially reduced. However, the actual AhR prevalence of active to inactive compounds among all chemicals is unknown, but various databases and model results indicate that there will be significantly fewer AhR active compounds than inactive compounds.^19,28^ Visualization of ToxCast database as a chemical space representing similarity of chemical structures (SW DataWarrior; Tanimoto similarity based on MDL keys (FragFp descriptors)^32^ showed structural overlaps between AhR-Inactive and Low-activity compounds, and between medium- and high-activity compounds (SM1-Figure S5). Compounds with low AhR activity often resemble inactive compounds, making them difficult to distinguish by using structural predictors. The overlap between compounds in Medium and High activity categories in our model could lead to an overestimation of activity levels, as Medium activity compounds were captured as active, but predicted to the High activity category (SM1-Table S4). In contrast, Low activity compounds could be undercaptured and hard to test due to their low specificity and sensitivity. Dividing the AhR activity into more than two categories of Active and Inactive compounds provides the advantage of predicting compounds with the highest activity but also indicates that most of the false active cases will be with low activity compounds. The model’s results are influenced by the database and descriptors used, and the database may not represent all compound groups equally, nor do the available predictors cover every structural aspect relevant for AhR activity. AhR can bind a wide variety of structurally dissimilar ligands, which is believed to result from differential binding of ligands within the AhR ligand-binding pocket, similarly to some members of the nuclear hormone superfamily like the pregnane X receptor.^50,61^

Regarding the model performance, our model for two (M_ALL 2) and three (M_ALL 3) AhR categories showed high sensitivity (90.1 and 83.6%) and specificity (83.7 and 80.4%) comparable to previously published QSAR models (Klimenko et al:^19^ sensitivity 85.1–89.1%, specificity 91.3–97.1%; Goya-Jorge et al.:^28^ sensitivity 0.71–0.76, specificity 0.77–0.82). Specificity (87.6%) was comparable also in the four-category model (M_arom 4), but the sensitivity (66.1%) was lower. However, no comparable multiclass QSAR models for the AhR activity are currently available. The use of different machine-learning (ML) algorithms (Adaboost, Random Forest, Gradient Boosting, Support-Vector Machine, and Multilayer Perceptron) resulted in model-performance differences within approximately 5%,^28^ suggesting that model accuracy depends more on the training-set quality and data curation than on the choice of ML algorithm.^42^ However, newer image-based approaches, such as DeepSnap-deep learning, may also improve the performance of QSAR models compared to traditional descriptor-based predictors.^62^

Although the random forest (RF) provides internal validation using oob samples (out-of-bag) to unbiasedly estimate the test set error, cross-validation is recommended for comparison with other techniques.^63^ However, when using both bootstrap validation (used internally by random forest) and cross-validation, the size of the cross-validation file needs to be taken into account, as the test set is selected twice. For models with three and four categories of AhR activity, the number of samples was not sufficient to perform cross-validation, and only bootstrap testing was performed. Our model was also validated for AhR active/inactive compounds using an external data set from Goya-Jorge et al.^28^ Our QSAR predictions matched their experimental results (accuracy 90%, SM2-Table A4), but only the classification into two AhR activity categories (Active/Inactive) could be verified, based on the details for experimental results provided by the authors. Additionally, predictions of our model marked as nonreliable, based on probability settings (Table 3), did not match the measured activity, and the accuracy of overall classification dropped from 90 to 72.5% after including the nonreliable predictions, highlighting the importance of setting the applicability domain and assessing result reliability.

QSAR models are reliable within their applicability domain (AD), but their accuracy decreases for compounds outside the chemical space of the training set.^64^ The Tanimoto distance is often used to measure the structural similarity of compounds with a threshold value (e.g., >0.4 or >0.6) used to define the AD.^19^ However, the use of the Tanimoto coefficient has certain limitations, particularly when it is applied to fingerprints that primarily capture 2D structural features. Additionally, setting an appropriate threshold to determine whether a compound lies outside the model’s structural applicability domain remains challenging. Moreover, the Tanimoto coefficient does not reflect the unreliability of the model in terms of misclassification of the compound due to the uncertainty of the structural predictors used.

For classification tasks, the model’s probability estimate can indicate prediction reliability, and for the random forest method, it is a suitable indicator of the applicability domain (AD),^39^ which includes both the reliability of structural similarity and classification based on the given structural predictors. Typically, the training data set is used to define the applicability domain, but the testing data set should also be considered.^63^ The sampling method in random forests is bootstrap, which is random sampling with replacement. As a result, most samples are used for both training and testing sets, independently across all trees in the forest.^65^

The underlying ToxCast database contains a wide range of chemicals, including pesticides, PPCPs, food additives, and industrial chemicals, which were selected on the basis of existing knowledge on toxicity, exposure potential, regulatory interest, and suitable properties for the automated high-throughput screening.^66^ The database contains many environmentally relevant contaminants; however, different compound groups are not evenly represented, and the applicability domain of the QSAR models built on the ToxCast data should always be carefully considered.^67^ The primary need for QSAR-based predictions was found for the metabolites and transformation products. Previous research has shown that QSAR models can reliably predict metabolite toxicity,^68^ making them a valuable tool for assessing the bioactivity of this understudied yet environmentally relevant group.^69,70^

### QSAR Model Prediction Used for Real-World Complex Environmental Mixture

Activation of AhR has been commonly induced by surface water samples, and several studies tried to link the observed effects to the detected compounds.^3,6,51,71^ These and other studies apply the concentration addition model as it has been shown to perform well for low-effect levels in mixtures^72^ and is generally recommended for chemicals acting through similar mechanisms or molecular pathways, such as endocrine-disrupting compounds.^73^ These studies covered extensive target lists, with the detected compounds generally contributing less than 10% of the observed activity. In the original study on the Danube River, the portion of explained effect was higher for the SR samplers (20–50%) than for HLB samplers (7–19%), except for site 5, where most of the effect could be explained. In this study, we aimed to increase the effect explicability by filling in the missing AhR potency data for all detected compounds, as potency data were unavailable for 164 compounds (36% of all detected compounds). This discrepancy between the SR and HLB samplers persisted even after applying the QSAR model and can be attributed to the distinct chemical profiles captured by the two sampler types. SR samplers preferentially accumulate nonpolar compounds, such as PAHs, legacy organochlorine pesticides, and brominated flame retardants, from larger water volumes. Owing to their aromatic ring structures, these compounds fall well within the model’s applicability domain, which also accounts for their comparatively higher predicted activities.

The developed QSAR model enabled the categorization of compounds based on their predicted AhR potency and prioritization for confirmatory experimental testing. Using this approach, we identified an additional 14 compounds potentially contributing to AhR potency, with experimental confirmation of activity for 7 of them. Two compounds, acridone and triphenylene, were indicated as candidate environmental AhR agonists and experimentally confirmed in this study for the first time. These compounds were found at every site, indicating widespread distribution in surface waters. Acridone is a transformation product of two antiepileptic drugs, carbamazepine (CBZ) and oxcarbazepine (OXC), which are commonly found in sewage and surface waters, formed through metabolism in the human body, and wastewater treatment processes.^74,75^ In addition, photodegradation of CBZ in surface waters also yields acridone, a metabolite exhibiting higher ecotoxicity than its parent drug.^76,77^ Triphenylene is a high molecular weight (HMW) PAH with four benzene rings, which occurs naturally in fossil fuels and can be found in tobacco smoke and engine exhaust. It is a ubiquitous product of incomplete combustion of coal, oil, and other organic compounds, and can be isolated from coal tar.^78^ Within the industry, triphenylene has increasing popularity in applications for discotic liquid crystal and electroluminescent materials.^79,80^ PAHs typically enter aquatic environments through both wet and dry atmospheric deposition, surface runoff, wastewater discharge, and industrial effluents. HMW PAHs are known to be insoluble in water and tend to bind to particulate matter, which facilitates their movement from soil and air into freshwater systems, as well as from the water surface to sediments.^81^

Importantly, the QSAR predictions not only prioritized the compounds with greater predicted AhR activity for confirmatory biotesting but also enabled filling a major gap in knowledge and taking into consideration the mixture effect modeling all detected compounds. Our results highlight the realistic situation when, despite filling the knowledge gaps in AhR potencies of all detected compounds, the drivers of a large part of the effect remain unknown at most sites. Further research applying methods, which enable identification of bioactive compounds outside the target analytical lists, i.e., effect-directed analysis or pull-down assays coupled to nontarget screening, is needed.^7,82,83^ The presented QSAR model and prioritization may be very useful in combination with these methods, as it can provide activity prediction for long lists of suspect structures, as it is not feasible to experimentally test these compounds, for which analytical standards are often very expensive or commercially unavailable.

## Conclusions

This study demonstrates the potential of multilevel QSAR models for identifying previously unknown AhR-active compounds and for achieving more accurate prioritization for experimental testing compared with binary (active/inactive) approaches. Multilevel QSARs also expand the applicability of in silico methods for estimating the toxicity of environmental mixtures and the contribution of individual chemicals to the overall toxicity. The results of AhR activity prediction also showed the importance of considering the applicability and reliability domains of the model and data curation. The cytotoxicity and low solubility of the compounds during experimental testing proved to be a problem, which prevented confirmation of the predicted activity of several compounds. Although the QSAR model demonstrated good predictive ability for compounds with high AhR activity (High, Medium-High, Medium), it showed a high percentage of false positives for compounds with low AhR activity, mainly due to structural overlap with inactive compounds. However, the splitting of the AhR activity into multiple levels allowed us to set more precise rules for prioritization of compounds both for testing and for assessing their potential contribution to the observed AhR activity of environmental samples. This approach also reduced the number of false positive results in the category of highly active compounds. Thus, when using QSAR models in real-world practice, it is necessary to consider limitations related to the quality and integrity of input data for the model and limitations in laboratory testing, such as cytotoxicity, low solubility, or measurement inaccuracies, which can significantly affect the verifiability of predictions.

## Supplementary Material





## Notes

Detailed information about data set curation for the QSAR model, prioritization and AhR activity calculation, in vitro bioassays, model performance and application, and comparisons with the Danish (Q)­SAR database and the literature (PDF)
A1: curated data set used for training QSAR models; A2: results of experimental testing; comparison of AhR activity predictions from the Danish (Q)­SAR database for compounds detected in the Danube River with the results of our QSAR model and available information from literature search; A3: calculations of the AhR effect explicability before and after the application of the QSAR model using two scenarios for 2 sampler types (HLB and SR samplers); and A4: comparison of results from experimental testing of compounds from Goya-Jorge et al. and predictions from our QSAR model in two categories of AhR activity (inactive/active) (XLSX)
