# Supplementary material for: Methodological Challenges in the Application of QSAR Models for Chemical Prioritization and Toxicity Assessment: A Case Study on Aryl Hydrocarbon Receptor Activity in Environmental Pollutant Mixtures
Source: ACS Environ Au. 2026 Jan 21;6(2):295–309. doi: 10.1021/acsenvironau.5c00224 (PMC13003357; doi:10.1021/acsenvironau.5c00224)
Supplement: Supplementary file 1 [file vg5c00224_si_001.pdf]

Supplementary Material 1 to:

Methodological Challenges in the Application of QSAR Models for Chemical Prioritization and Toxicity Assessment: A Case Study on Aryl Hydrocarbon Receptor Activity in Environmental Pollutant Mixtures

Jiří Komprda[1], Katarína Lörinczová[1], Zuzana Toušová[1], Marie Smutná[1], Soňa Smetanová[1], Klára Komprdová[1], Klára Hilscherová[1]**

[1]RECETOX, Faculty of Science, Masaryk University, Kotlarska 2, Brno, 602 00, Czech Republic.

Contents

Section S1. Dataset curation for QSAR model 1

Section S2. Dataset for prioritization and calculation of AhR activity 4

S2.1. Priority dataset 4

S2.2 Linking of AhR activity to target chemicals with QSAR model predictions and experimental data 6

Section S3. *In vitro* bioassay to assess AhR potency 6

Section S4. Results of the dataset curation for QSAR model 7

Section S5. Molecular descriptors 8

Section S6. Model parametrization and validation 10

Section S7. Comparison of prediction results with the Danish (Q)SAR database and literature 13

S7.1. Danish (Q)SAR database 13

S7.2. Comparison with literature 14

Section S8. QSAR model application 14

Section S9. Cytotoxicity 15

References 16

Abbreviations:

A, Active; AhR, Arylhydrocarbon receptor; BEQ, Bioanalytical Equivalent Concentration; BEQbio, Bioanalytical equivalent based on *in vitro* testing; BEQchem, Bioanalytical equivalent based on chemical analyses; CAS, Chemical Abstracts Service; DMEM, Dulbecco's Modified Eagle Medium; EC_{25} / EC_{20}, Effect concentration reaching 25%/20% of the maximum effect response relative to the response of standard reference compound; FA, False Active; FBS, Fetal Bovine Serum; FI, False Inactive; GFP, Green Fluorescent Protein; HLB, AttractSPETM HLB disks = Hydrophilic Lipophilic-Balanced sorbent; I, Inactive; JDS4, Joint Danube Survey 4; MD, Missing Data; MDL, Molecular Design Limited (company); OA, Overall Accuracy; PAHs, Polycyclic Aromatic Hydrocarbons; PBDEs, Polybrominated Diphenyl Ethers; PBS, Phosphate Buffer Saline; PCBs, Polychlorinated Biphenyls; PCDDs, Polychlorinated Dibenzo-p-dioxins; PCDFs, Polychlorinated Dibenzofurans; PPCPs, Pharmaceuticals and Personal Care Products; PPV, Positive Predictive Value; qHTS, quantitative High-throughput Screening; QSAR, Quantitative Structure–Activity Relationship (QSAR); REP, Relative effect potency; RF, Random forest; SR, Silicone Rubber; TA, True Active; TCDD, 2,3,7,8-Tetrachlorodibenzo-p-dioxin; TI, True Inactive.

Section S1. Dataset curation for QSAR model

Stepwise curation of the raw datasets (**Table S1**) was carried out according to a workflow shown in **Figure S1**.

1. All datapoints (corresponding to individual experiments with unique sample ID) in the Datasets 2 and 4 with either unreported or insufficient compound purity (<90%) in Dataset 1 were excluded. Mixtures were removed in this step.
2. Effect concentrations (EC_{20} and EC_{25} for cell viability and AhR agonism, respectively) were calculated for each datapoint in Dataset 2 and Dataset 4 using parameters from the winning curve-fitting model in Comptox (Hill or Gain-Loss model) according to **Equation (1)**, where *modl_tp* is winning model top of curve; *modl_gw* is winning model gain slope; and *modl_ga* is winning model gain.

$$EC_x = \left(-\frac{\log\left(\frac{modl\_tp}{x}-1\right)}{modl\_gw - modl\_ga}\right)^{10} \qquad \text{Equation (1)}$$

   Calculated EC_{20} for cell viability was defined as a cut-off to establish the cytotoxicity limit. Compounds with missing Hill parameters or not reaching the effect of 20% on cell viability were considered as non-cytotoxic within the tested range (<1 000µM). The EC_{25} for AhR agonism was defined as the cut-off for the magnitude of AhR potency similarly to Nikolov et al., (2023) and to clearly distinguish between 4 activity categories (inactive with EC_{25}>1 000 µM; low with EC_{25} range 11-100 µM; medium with EC_{25} range 2.03-10 µM; and high EC_{25} <2.03 µM). Arithmetic means of EC_{20} and EC_{25} values for cell viability and AhR agonism, respectively, were calculated for compounds with multiple experimental datapoints available. Mean values were used for further curation steps and activity categorization.

3. To account for cytotoxicity, compounds were excluded from the final dataset if their mean EC_{25} for AhR agonism (from Dataset 4), when multiplied by a factor of three, exceeded the mean EC_{20} for cell viability, based on Dataset 2 or, if unavailable, Judson's viability data from Dataset 3. In addition, compounds with EC_{20} for specific cell viability (Dataset 2) or Judson's viability (Dataset 3, if missing in Dataset 2) lower than 100 µM and inactive on AhR agonism (EC_{25}>1 000µM) were also excluded.
4. In the set of AhR inactive compounds (EC_{25}>1 000µM), structures with undefined CAS registry numbers were removed.
5. In the set of AhR active compounds, all structures with inconsistent responses in repeated experiments were removed from the final dataset as well as all structures with very low potency (EC_{25}>100µM), which were considered inconclusive for the QSAR model.
6. In the set of AhR active compounds in categories 2 and 3 with medium and high potencies, all dose-response curves of AhR agonism and cell viability were manually checked and structures with atypical curve shapes were removed from the final dataset. Decision on compounds' removal in this step was based on subjective expert judgement.
7. Possible interference of the compounds with the luciferase readout was checked against data from **tox21-luc-biochem-p1,** which is a qHTS assay to identify small molecule inhibitors of firefly luciferase. None of the structures showed any interference and thus this curation step is not included in the overall scheme in **Figure S1**.

Table S1. Overview of the datasets used for data curation and compiling the final dataset of reliable effect data on AhR potency to build the QSAR model

	**Dataset content/ Assay endpoints**	**Identification of the data source**	**Link**
**Dataset 1**	Compound purity	tox21-ahr-p1	https://tripod.nih.gov/pubdata/index.html
**Dataset 2**	Specific cell viability	TOX21_AhR_LUC_Agonist_viability (assay ID 807)	https://comptox.epa.gov/dashboard/assay-endpoints/807
**Dataset 3**	Non-specific cell viability	cell viability/cytotoxicity based on Judson et al., (2016)	https://academic.oup.com/toxsci/article/152/2/323/2578946
**Dataset 4**	AhR agonism	TOX21_AhR_LUC_Agonist (assay ID 806)	https://comptox.epa.gov/dashboard/assay-endpoints/806

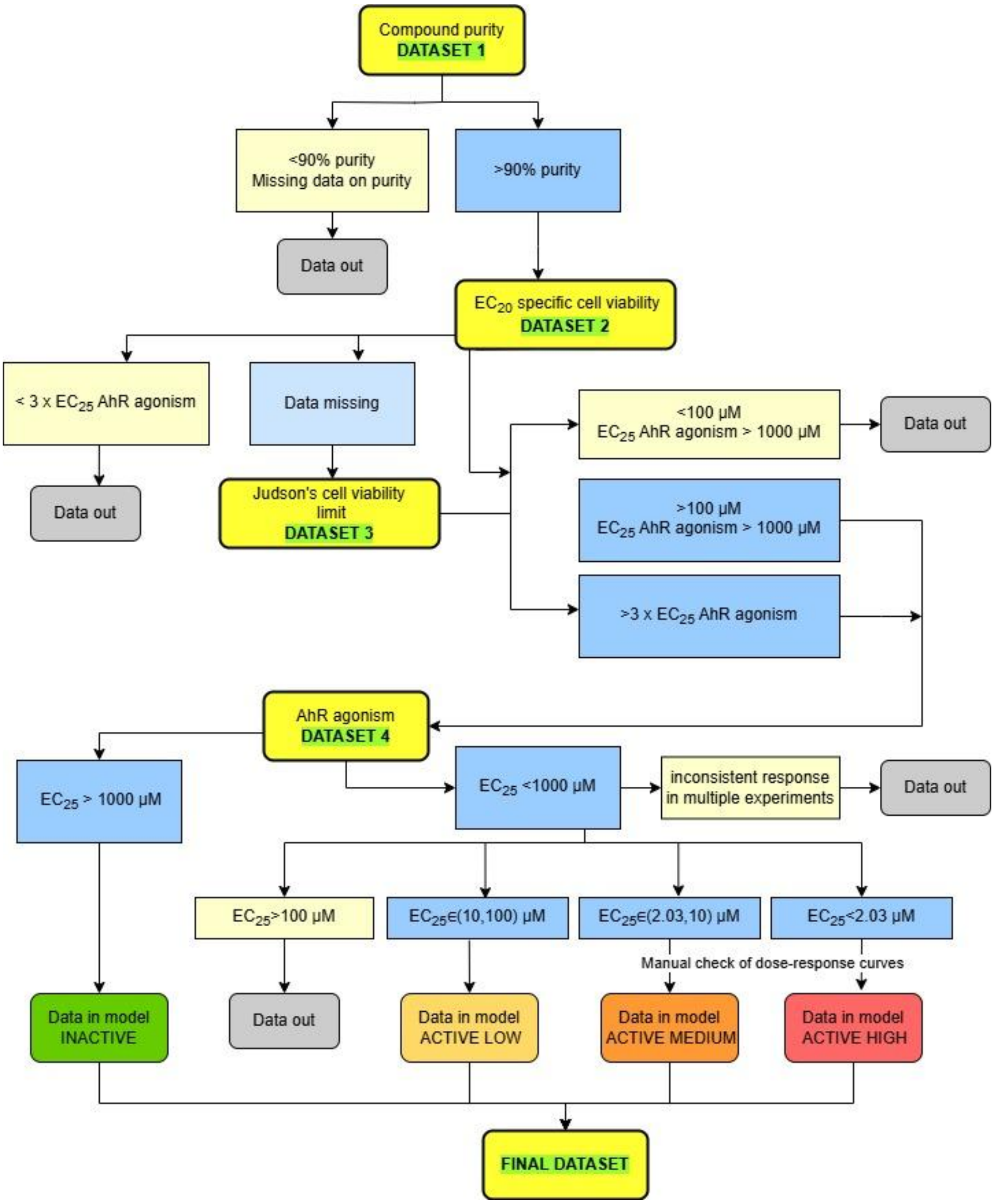

Figure S1. Overview of the data curation pipeline

Section S2. Dataset for prioritization and calculation of AhR activity

S2.1. Priority dataset

The prediction of AhR activity was applied to a subset of targeted compounds (N=164) that were analysed within the Joint Danube Survey 4 (JDS4), an extensive monitoring effort along the Danube River [1]. The surface water extracts obtained by long-term passive sampling employing silicone rubber (SR) sheets for hydrophobic compounds and AttractSPE^{TM} HLB disks for hydrophilic compounds from 9 locations were subjected to chemical analyses and effect-based assessment (**Figure S2**). The monitored contaminants spanned multiple compound classes including pesticides, industrial compounds, pharmaceuticals and personal care products (PPCPs) and metabolites, polycyclic aromatic hydrocarbons (PAHs), polychlorinated biphenyls (PCBs), and polybrominated diphenyl ethers (PBDEs). Effect potencies of passive samplers' extracts were determined using *in vitro* assays addressing several cellular endpoints including the AhR-mediated activity, which was detected and quantified at all sampling sites in both sampler types. Iceberg modelling using the bioanalytical equivalent concentration (BEQ) approach was utilized to determine the contribution of detected compounds to the observed effect. The BEQ of each detected compound (BEQ_{chem}) was estimated by multiplying its concentration (c_i) and the relative effect potency (REP) value (Neale et al., 2020). The study by Šauer et al.[1] addressed the total of 747 analytes and the information on their potencies to activate the AhR was retrieved from the CompTox database (https://comptox.epa.gov/dashboard/; version 2.1.1; data downloaded in August 2022). The effect potencies (REPs) relative to standard reference compound 2,3,7,8-Tetrachlorodibenzo-p-dioxin (TCDD) were calculated according to **Equation (2)** for each target compound available in the Comptox database (Williams et al., 2017), where EC_{25} refers to effect concentration causing 25% of the maximal response.

$$REP_{25} = \frac{EC_{25}(TCDD)}{EC_{25}(compound)} \qquad \text{Equation (2)}$$

The total BEQ_{chem} based on chemical analyses was calculated as a sum of individual BEQ_{chem} values of all compounds with available REP detected at the particular site (**Equation (3)**) following the concept of concentration addition [2].

$$BEQ_{chem} = \sum_{i=1}^{n} REP_i \times c_i \qquad \text{Equation (3)}$$

The relative contribution of detected compounds to the observed AhR potencies of environmental extracts determined with an *in vitro* bioassay (BEQ_{bio}) was calculated as a ratio of BEQ_{chem} and BEQ_{bio} expressed in percent.

Of the detected compounds (N=456; 61%) with available REP (N=292), the total of 53 active chemicals contributed to the observed AhR effect. On average, these compounds accounted for 23% and 37% of the bioactivity in HLB and SR samplers, respectively. Sampling site 5 was an exception, as the observed AhR effect could largely be attributed to the detected chemicals [1]. The list of 164 compounds from the Danube case study, which were detected, but their AhR potency was unknown (yellow box in **Figure S3**), was established as a realistic environmental dataset for application of the newly developed QSAR model and prioritization scheme. Predicted potencies were used to calculate the relative contribution of the 164 detected compounds and a subset of chemicals was prioritized for experimental validation of the model in the *in vitro* bioassay.

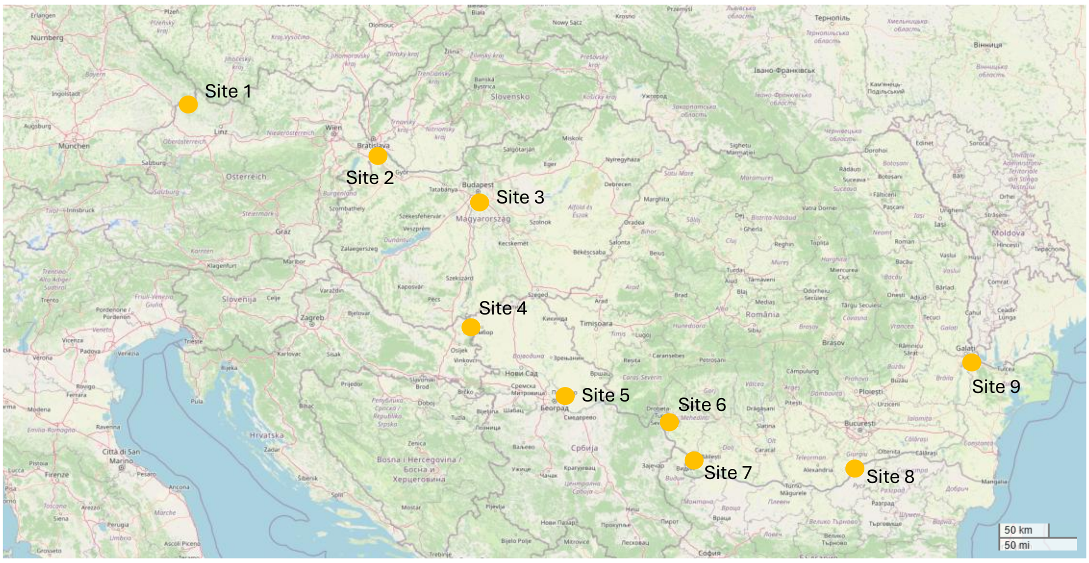

Figure S2. Map of sampling sites on the Danube River. Samples were collected by long-term passive sampling within the Joint Danube Survey 4 monitoring campaign in 2019[1]. Created by authors in OpenStreetMap (*www.openstreetmap.org*).

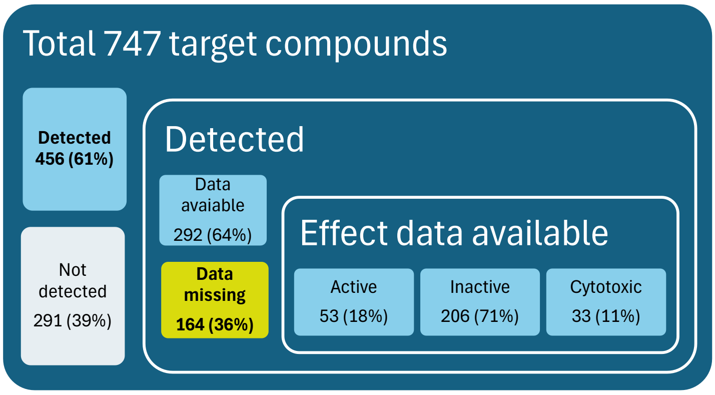

Figure S3. Overview of the results from targeted chemical analysis in the Danube case study [1]and availability of the effect data for AhR activation in the CompTox database (version 2.1.1; downloaded 2022).

S2.2 Linking of AhR activity to target chemicals with QSAR model predictions and experimental data

To determine the total BEQ_{chem} for AhR activity, to sum of REP_{EC25} values of individual compounds multiplied by their measured was calculated for extracts from individual sites and samplers on the River Danube as explained above. REP_{EC25} values based on ToxCast were used for compounds with previously known AhR activity (N=292). The missing REP_{EC25} values for detected compounds with unknown AhR potency (N=164) were predicted by the newly developed QSAR model. The predicted REP_{EC25} values were determined based on the predicted activity category as the geometric mean of the EC_{25} value of ToxCast compounds within the category. In case of the compounds prioritized for experimental AhR activity validation, the REP_{EC25} values were calculated from the Caflux bioassay results (mean EC_{25} value) except for Cyclopenta[cd]pyrene, for which experimental REP_{EC25} value was taken from a study by Machala et al.,[3]. REP_{EC25} values were not determined for cytotoxic compounds as well as for compounds with unreliable predictions (see **Section S4**) and thus were not used for the overall BEQ_{chem} calculations.

The site-specific contribution of compounds to the observed AhR activity was calculated according to various QSAR model application methods (scenarios), where reliable activity predictions were used with different data sources for the calculation of the REP_{EC25} values. Detailed description of the scenarios can be found in **Section S4** and in **Table S6**. For each sampling site, the overall AhR activity (BEQ_{chem}) was calculated and the number of compounds contributing to the bioactivity was determined for both samplers (HLB and SR). The explicability of the observed AhR activity on the Danube River was compared before and after the QSAR model application.

Section S3. *In vitro* bioassay to assess AhR potency

AhR-mediated (dioxin-like) activity of prioritized compounds suspected of AhR potency was assessed using Caflux cell line derived from mouse hepatoma stably transfected with AhR-responsive green fluorescent protein (GFP) reporter (H1G1.1c3) [4] as described in Šauer et al. [1]. The cells were routinely cultivated in a humidified incubator with 5 % CO_2 in tissue culture flasks (TPP, Austria) and white DMEM medium, supplemented with 10 % fetal bovine serum (FBS), (Sigma-Aldrich, Czech Republic). The bioassay was performed in black 96-well plates, and the reference compound TCDD, tested chemicals or solvent (solvent control) were added 24h after seeding of the cells in optimal density (20, 000 cells per well). After another 24h of exposure, the fluorescence (485/520) was measured following the removal of the medium and washing the cells with PBS (Phosphate buffer saline). Each treatment was tested in triplicate and each compound was tested in a dilution series to describe the dose-response

relationship in at least two independent experiments. The effect level of tested compounds was normalized to the dose-response calibration of TCDD. The dose-response data were evaluated with GraphPad Prism 9 software (GraphPad® Software, San Diego, California, USA) using the Hill model for non-linear regression to estimate the EC_{25}. REP values of tested compounds were calculated according to Equation 2.

Cell viability was assessed using confluency as a surrogate for the number of cells in each well using Cytation 5 Cell Imaging Multi-Mode Reader (equipped with 4 × objective and Gen5 Image Prime 3.10.06 software, BioTek). Treatments with relative confluence lower than 90 % of the solvent control were considered cytotoxic and excluded from calculations of EC_{25}.

Section S4. Results of the dataset curation for QSAR model

From a dataset of 7 905 ToxCast compounds with molecular descriptors, a total of 3 022 (38%) structures were excluded during successive data curation steps. Specifically, 1 928 compounds (24.4%) were excluded due to insufficient purity, 860 compounds (10.9%) due to cytotoxicity, 130 compounds (1.6%) due to low activity (EC_{25} > 100 µM), 46 compounds (0.6%) due to inconsistent results from repeated measurements, and 58 compounds (0.7%) were manually excluded due to poor-quality dose-response curves. The distribution of excluded compounds across different AhR activity categories is provided in **Table S2**. The proportion of inactive versus active compounds in the final dataset (10.4 times more inactive compounds) is slightly higher than in the non-curated dataset (7.3 times more inactive compounds). The curation process aimed to remove compounds with unreliable AhR activity and retain only those with confirmed activity in the final dataset.

Table S2. Overview the data curation and resulting numbers of excluded compounds across different AhR activity categories in particular curation steps.

	**Total**	**Inactive**	**Active**		**Very low** EC_{25} > 100 µM	**Low** EC_{25} 11-100 µM	**Medium** EC_{25} 2-10 µM	**High** EC_{25} < 2 µM
**Compounds for model**	4 883 (62%)	4 457 (56%)	426 (5.4%)	→		276 (3.5%)	103 (1.3%)	47 (0.59%)
							**Medium-High** EC_{25} <11 µM 150 (1.9%)	
**Excluded compounds**	3 022 (38%)	2 499 (32%)	523 (6.6%)	→	197 (2.5%)	228 (2.9%)	61 (0.77%)	37 (0.47%)
**Impure**	1 928 (24%)	1 756 (22%)	172 (2.2%)	→	32 (0.40%)	93 (1.2%)	27 (0.34%)	20 (0.25%)
**Cytotoxic**	860 (11%)	723 (9.2%)	137 (1.7%)	→	34 (0.43%)	87 (1.1%)	13 (0.16%)	3 (0.04%)
**Low activity**	130 (1.6%)		130 (1.6%)	→	130 (1.6%)			
**Inconsistent data**	46 (0.58%)		46 (0.58%)	→	0	45 (0.57%)	0	1(0.01%)
**Manually excluded**	58 (0.73%)	20 (0.25%)	38 (0.48%)	→	1 (0.01%)	3 (0.04%)	21 (0.27%)	13 (0.16%)

Section S5. Molecular descriptors

Despite extensive computational efforts to elucidate the AhR signaling pathway, the absence of experimentally determined structures, particularly the ligand-binding (PAS-B) domain, along with several unresolved molecular mechanisms, still hinder a comprehensive description of the AhR structure and AhR ligand-binding. It also remains unclear whether diverse ligands, binding in different ways within the ligand-binding cavity, can differently influence individual steps of the AhR pathway[5,6]. Therefore, the most relevant descriptors identified in our analysis highlight the structural features most likely involved in the AhR binding mechanisms, although their direct association with the structurally elusive PAS-B domain cannot yet be confirmed.

Results of models show that the most important descriptors are from group of 3D-MoRSE molecular structural descriptors. These descriptors are derived from 3D atomic coordinates using a transformed distance matrix to capture the molecule's shape and spatial distribution of atoms. 3D MoRSE descriptors can clearly distinguish between active and non-active compounds on very good level of significancy (Figure S4). Li et al. [7] found that Mor14u descriptor belongs to most significant descriptors for a large dataset containing PCBs, PCDDs and PCDFs. In our study most important 3D MoRSE descriptors are Mor23m and Mor12v where "m" denotes weighting by atomic mass and "v" denotes weighting by atomic van der Waals volume. Parameters 23 and 12 represent discrete values of the scattering angle (or resolution) used in the mathematical transform simulating X-ray/electron diffraction, effectively controlling the spatial frequency at which the molecular 3D structure is probed. Mor23m reflects global 3D shape (long distance interactions) and possible heavy-atom distribution (presence of halogens), in contrast Mor12v indicates importance of local steric effects (short distance interaction) such as ortho/para substitution. Other important descriptors generally indicate importance of lipophilicity (log Kow), presence of aromatic rings of size 6, rigidity and compactness (number of rotatable bonds). Small number of rotatable bonds indicates that molecule has stable 3D shape, supports rigid, planar conformations.

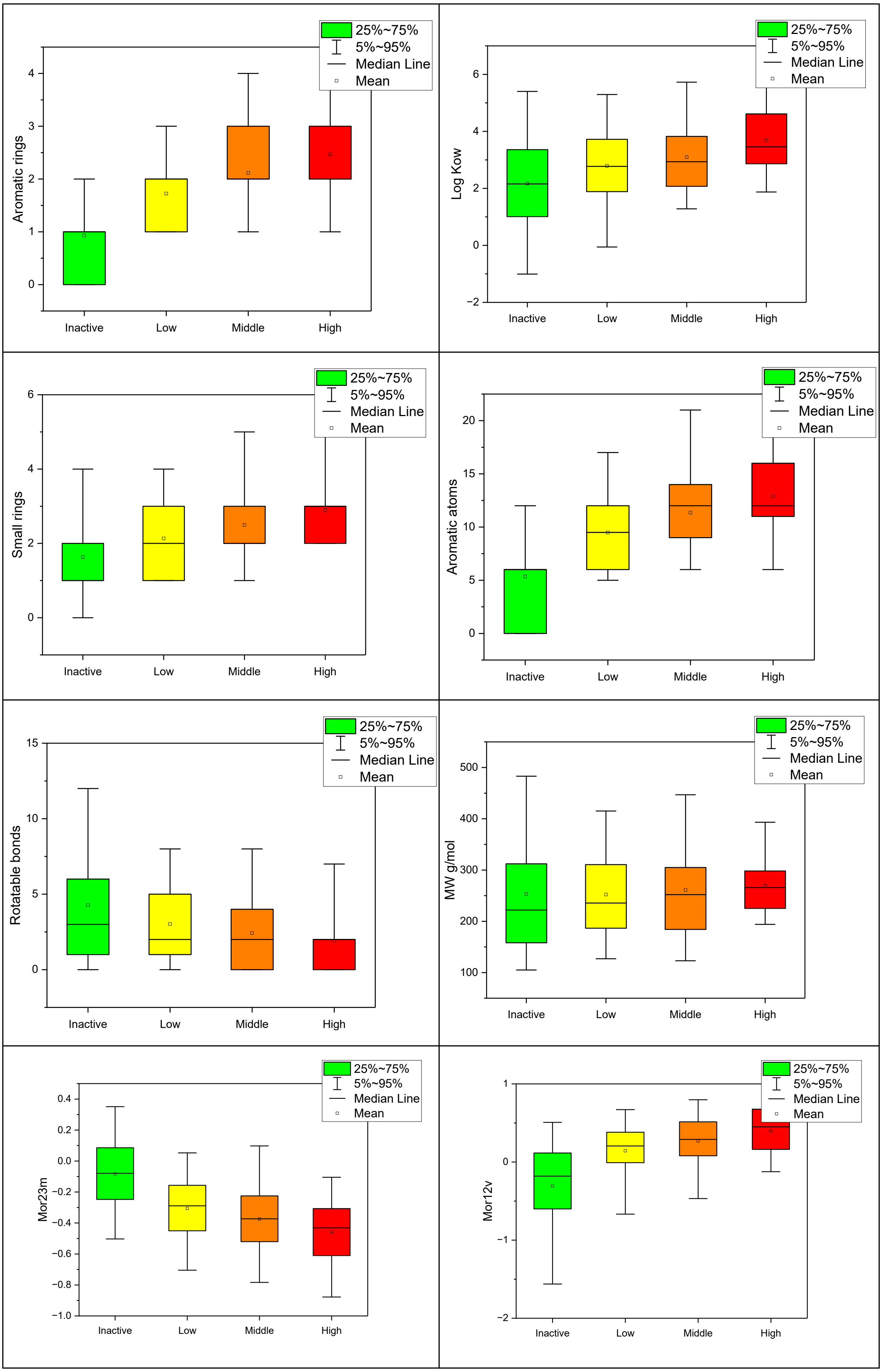
25%~75%
5%~95%
Median Line
Mean
Aromatic rings
Log Kow
Small rings
Aromatic atoms
Rotatable bonds
MW g/mol
Mor23m
Mor12v
Inactive
Low
Middle
High

Figure S4. Distribution of compounds from the curated CompTox dataset within four AhR activity categories according to selected molecular descriptors.

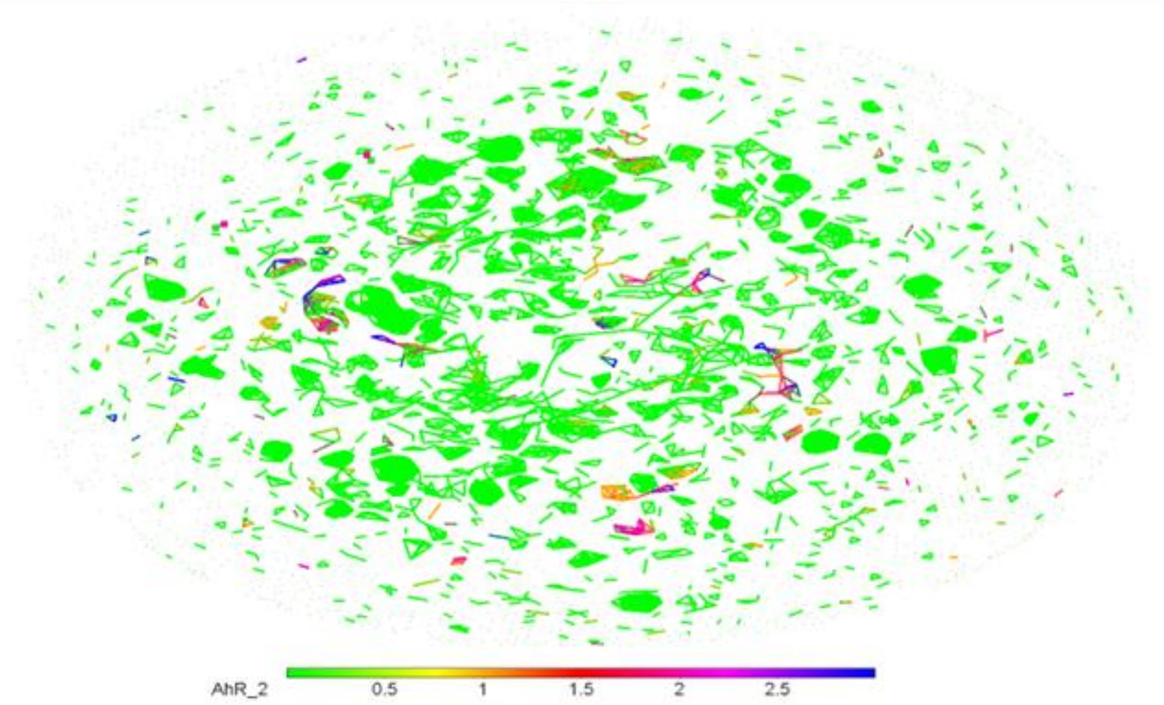

Figure S5. Visualization of chemical space concerning structural similarity of chemical compounds based on MDL keys in curated dataset (DataWarrior SALI Plot). It shows clusters ("islands") of compounds grouped by structural similarity and colors related to their AhR activity (green means Inactive, yellow and orange means Low activity, red and purple means Medium and blue means High activity).

Section S6. Model parametrization and validation

All model hyperparameter settings, including the optimal parameter values for the final model, are provided in the Table S3. Model parameters from Table S3 were tested using a step size of 0.1 for weights, a step size of 1 for the number of predictors and number of samples in terminal nodes, and a step size of 100 for the number of trees. Bayesian optimization of parameters for each combination of weights has been run. The optimal weighting of individual categories was selected based on the model with the highest WOA. The difference in overall accuracy (OA) between the test and training sets should be no more than 5% to 10%. Slightly worse results were obtained for the multi-category model because the optimization procedure is generally set for overall accuracy and not weighted, thus in favour of categories with more observations, which was partially corrected by the weights of each category. Even reducing the size (e.g. number of nodes) of the trees did not lead to smaller differences between the test and training sets.

Table S3. Hyperparameter optimisation for Random Forest models.

		Hyperparameter RF			Overall accuracy	
AhR activity categories	Model	Hyperparameter RF*	Tested range	Optimal value	OA (testing)	OA (training)
Inactive/Active	M_ALL 2<br><br>All substances	number of predictors	2-60	52	84.2%	85.9%
		number of samples in terminal nodes	2-30	20		
		number of trees	500-2 000	1 000		
		weights	0.8-1; 1-2.5	1,2		
Inactive/Active	M_arom 2<br><br>Aromatic substances	number of predictors	2-60	27	76.1%	83.4%
		number of samples in terminal nodes	2-30	20		
		number of trees	500-2 000	1 000		
		weights	0.8-1; 1-2.5	1,2		
Inactive/Low/ Medium-High	M_ALL 3<br><br>All substances	number of predictors	2-60	60	79.2%	84.6%
		number of samples in terminal nodes	2-30	21		
		number of trees	500-2 000	1 000		
		weights	0.8-1; 1-2; 1-3	1, 1.2, 1.5		
Inactive/Low/ Medium-High	M_arom 3<br><br>Substances with aromatic ring	number of predictors	2-50	35	85.4%	93%
		number of samples in terminal nodes	2-30	5		
		number of trees	500-2 000	1 000		
		weights	0.8-1; 1-2; 1-3	1,1.2, 1.6		
Inactive/Low/ Medium/High	M_arom 4<br><br>Substances with aromatic ring	number of predictors	2-50	32	81.9%	92.3%
		number of samples in terminal nodes	2-30	5		
		number of trees	500-2 000	1 000		
		weights	0.8-1; 1-2; 1-2; 1-3	1,1.2,1.5, 2		

*The ranges/values for the weights of the AhR activity categories are in the order: Inactive/Active for models with two activity categories, Inactive/Low/Medium-High for models with three activity categories and Inactive/Low/Medium/High for model with four activity categories.

Table S4. Results of classification into three and four categories of AhR activity on the oob test set.

M_ALL 3			Predicted class (numbers;percentages)				Sensitivity/Accuracy		Specificity	Precision	FA
			Inactive	Low	Medium-High	Total N	class	I/A			
All substances	True class	Inactive	3 585<br>80.4%	733<br>16.5%	139<br>3.1%	4 457	80.4%	80.4%	85.9%	98.1%	
		Low	70<br>25.4%	131<br>47.5%	75<br>27.2%	276	47.5%	74.6%	84.1%	15.2%	84.8%
		M+H	0<br>0%	0<br>0%	150<br>100%	150	100%	100%	94.0%	41.2%	38.2%
		Total N	3 655	864	364	4883	*76%	85%			

M_arom 3			Predicted class (numbers; percentages)				Sensitivity/Accuracy		Specificity	Precision	FA
			Inactive	Low	Medium-High	Total N	class	I/A			
Substances with aromatic ring	True class	Inactive	2 372<br>90%	210<br>8%	55<br>2.1%	2 637	90%	90%	74.8%	94.6%	
		Low	127<br>49.4%	94<br>36.6%	36<br>14%	257	36.6%	50.6%	92.2%	30.3%	67.7%
		M+H	9<br>6.2%	6<br>4.1%	131<br>89.7%	146	89.7%	93.8%	93.8%	59.0%	24.8%
		Total N	2 508	310	222	3 040	*72.1%	78.1%			

M_arom 4			Predicted class (numbers; percentages)					Sensitivity/Accuracy		Specificity	Precision	FA
			Inactive	Low	Medium	High	Total N	class	I/A			
Substances with aromatic ring	True class	Inactive	2 309<br>87.6%	270<br>10.2%	43<br>1.6%	15<br>0.6%	2 637	87.6%	87.6%	69.2%	94.9%	
		Low	111<br>43.2%	114<br>44.4%	26<br>10.1%	6<br>2.3%	257	44.4%	56.8%	89.8%	28.7%	68%
		Medium	13<br>13.1%	13<br>13.1%	19<br>19.2%	54<br>54.6%	99	19.2%	86.9%	97.9%	21.6%	48.9%
		High	0<br>0%	0<br>0%	0<br>0%	47<br>100%	47	100%	100%	97.5%	38.5%	12.3%
		Total N	2 433	397	88	122	3 040	*62.8%	82.2%			

M+H (Medium-High), A (Active), I (Inactive), FA (False activity, model predicts inactivity for active chemicals), TA (true active), TI (true inactive), FI (false inactive), Overall accuracy (OA)=(TA+TI)/(TA+TI+FI+FA), Sensitivity=TA/(TA+FI), Specificity=TI/(TI+FA), Precision(PPV, Positive predictive values)=TA/(TA+FA), Negative predictive value (NPV)=TI/(TI+FI), *Weighted overall accuracy (WOA) = (sum of class Accuracy)/n class,

Section S7. Comparison of prediction results with the Danish (Q)SAR database and literature

S7.1. Danish (Q)SAR database

Table S5. Comparison of AhR activity prediction results for prioritized substances detected in the Danube River, as predicted by the QSAR model (M_ALL 2) developed in this study, with predictions from the Danish (Q)SAR Database (http://qsar.food.dtu.dk).

**N=164**		**The Danish (Q)SAR database**				
		POS_IN	NEG_IN	OUT	MD	Summary
		**Rational final model**				
**Our QSAR model (M_ALL 2)**	Active	0	0	13	1	14
	Inactive	1	50	24	31	106
	OUT	2	5	24	13	44
	Summary	3	55	61	45	164
		**Random final model**				
	Active	4	0	9	1	14
	Inactive	0	49	26	31	106
	OUT	7	6	18	13	44
	Summary	11	55	53	45	164

POS_IN (positive (active) prediction inside applicability domain), NEG_IN (negative (inactive) prediction inside applicability domain), OUT (prediction outside applicability domain), MD (Missing data)

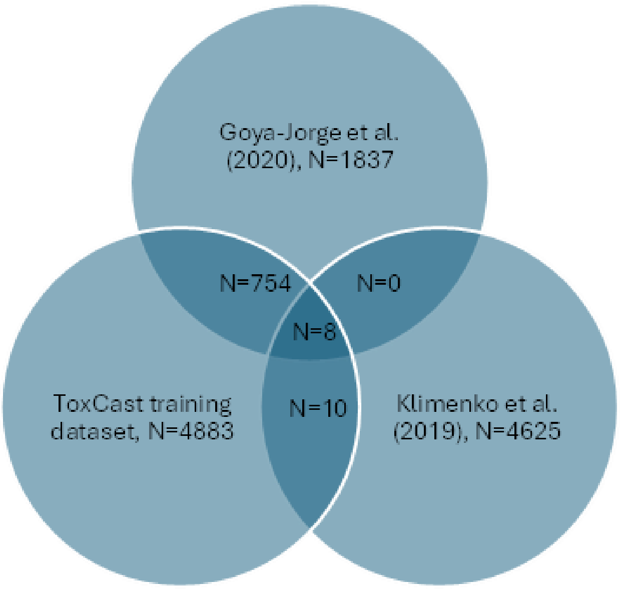

Figure S6. Overlaps among databases used for QSAR models of AhR activity (exclusive intersections). ToxCast training dataset was used in this study.

S7.2. Comparison with literature

In vitro AhR activity for all detected compounds with unknown AhR potency was searched in scientific literature and the results are provided in SM2-A2. A total of 44 out of 164 compounds with non-reliable model predictions were excluded from the comparison and only compounds with reliable model predictions were considered (N=120). For 78 out of 120 compounds, experimental data from literature was missing. Literature data on AhR activity were available for 42 compounds. For comparison, only *in vitro* proven AhR agonistic activity and inactivity was taken into consideration (N=28).

AhR active agonists (N=7) identified in literature included 5 polycyclic aromatic hydrocarbons (PAHs) and 2 pharmaceuticals and personal care products (PPCPs). AhR active predictions by our model were in concordance for all AhR active agonists (5 High active, 1 Medium-High active, 1 Low active). AhR inactives in literature (N=21) consisted of various compounds such as PPCPs (N=10), pesticides (N=4), insecticides (N=4), PAHs (N=1) and 2 industrial compounds (1 plastic additive and 1 surfactant). All of them were also predicted AhR inactive by our model except for Perylene (High activity prediction), where its activity was also confirmed experimentally. Furthermore, 14 compounds were found to be also experimentally active either as AhR antagonists (N=2) or with unspecified AhR activity (N=12) in different literature resources (SM2-A2).

Section S8. QSAR model application

For QSAR model application, predictions from two developed QSAR models (M_arom 4 model and M_ALL 3 model) were combined to determine the contribution of the prioritized compounds to the overall AhR activity. In general, two application strategies were applied to generate REP_{EC25} values (**Table S6**) based on reliable model predictions with different data sources for the calculation of the REP_{EC25} values (Scenario 1 and Scenario 2). In Scenario 1, for all detected compounds with unknown AhR potency, REP_{EC25} values were calculated from predicted AhR activity (geometric mean of the EC_{25} value of the AhR activity category). In Scenario 2, in case of compounds experimentally tested *in vitro*, REP_{EC25} values were calculated from EC_{25} value obtained in the Caflux bioassay (mean EC_{25} value) instead of model prediction.

Table S6. QSAR model application strategy for the calculation of prioritized compounds' contribution to the overall AhR activity

Application method	Data source for the REP_{EC25} value calculation
**Scenario 1**	Model prediction (geometric mean of EC_{25} value of the activity category from the CompTox dataset)
**Scenario 2**	Experimental testing results, if available (mean EC_{25} value); otherwise model prediction (geometric mean of EC_{25} value of the activity category)

Section S9. Cytotoxicity

The experimental validation of predicted AhR activity of prioritized compounds was compromised with frequent detection of cytotoxicity within the tested concentration range. For citalopram (antidepressant), propranolol (antihypertensive) and pethoxamid (herbicide), we observed cytotoxicity limits higher than 50µM and no AhR activity up to this level. These compounds are likely AhR inactive. If these compounds were low agonists (with EC_{25} higher than 50µM), their contribution to the observed AhR activity would be negligible given their relatively low measured concentrations. The cytotoxicity detected at lower concentrations seen while testing the AhR activity of PhIP (food contaminant), harmine and harmane (alkaloids found in food, coffee or tobacco smoke), sertraline (antidepressant), duloxetine (antidepressant) and metazachlor (herbicide), could possibly have masked their activity at higher than cytotoxic concentrations. In such case harman, a heterocycylic aromatic amine detected at all sites, with low predicted AhR activity, could contribute a non-negligible portion of the observed AhR activity due to its relatively high environmental concentrations. Similarly, PhIP and harmine (detected at 3 and all sites, respectively) structurally related to harman, could contribute another small portion of the effect if these compounds had EC_{25} higher than the observed cytotoxicity. This is not reflected in the presented results of Scenario 2 in Figure 3 in the main manuscript, where all the cytotoxic compounds were excluded from the calculations.

Cytotoxicity at low concentrations of the above-mentioned compounds was reported earlier in literature and is not unusual for cancer cell lines typically used in in vitro research studies. PhIP was reported as cytotoxic at 5µM in CaCo and HepG2 cells by Rogers et al. [8]. Cytotoxicities of citalopram at 10µM (EC_{20}) and sertraline at 5µM (EC_{20}) were reported in cancer cells in a study by Bin Kanner et al. [9]. Cytotoxicity of duloxetine of 17.1µM and 19.6µM was reported for HeLa and MDA-MB-231 cells, respectively in a study by Nikolic et al. [10]. Cytotoxicity of metazachlor reaching 36µM in HepG2 was reported by Ferguson et al. [11]. The cytotoxicity of propranolol was ranged from 75-125 µM in sarcoma

cells [12]. Pethoxamid elicited cytotoxicity in HepG2 cells at 3.71 µM [13]. In case of harman and harmine, the literature reported cytotoxicity values (100µM and 25µM, respectively) were higher than the levels observed in our study [14,15].

Cytotoxicity complicates experimental validation of specific modes of action across various *in vitro* assays, and this issue was pronounced in our study. Incorporating a complementary cytotoxicity model and using its predictions to further filter compounds in prioritization schemes could be a promising strategy that should be further examined, namely in case where extensive dataset with dose-response data for cytotoxicity of wide spectrum of chemicals for the specific cell line are available. However, while cytotoxicity can be influenced by structural features such as lipophilicity, hydrogen bonding capacity, molecular size, and electronic properties, that often appear in QSAR models for cytotoxicity prediction, it is a very complex endpoint involving multiple mechanisms (e.g., oxidative stress, mitochondrial dysfunction, DNA damage). Therefore, it can be rather non-specific with respect to molecular structure and not tied to a single structural motif [16]. This complexity limits the applicability domain of QSAR models for cytotoxicity and often requires large, diverse datasets and advanced algorithms to capture pattern. Therefore, the limitations of such cytotoxicity models must also be carefully considered.

References

(1) Šauer, P.; Vrana, B.; Escher, B. I.; Grabic, R.; Toušová, Z.; Krauss, M.; von der Ohe, P. C.; König, M.; Grabicová, K.; Mikušová, P.; Prokeš, R.; Sobotka, J.; Fialová, P.; Novák, J.; Brack, W.; Hilscherová, K. Bioanalytical and Chemical Characterization of Organic Micropollutant Mixtures in Long-Term Exposed Passive Samplers from the Joint Danube Survey 4: Setting a Baseline for Water Quality Monitoring. *Environment International* **2023**, *178*, 107957. https://doi.org/10.1016/j.envint.2023.107957.

(2) Escher, B.; Braun, G.; Zarfl, C. Exploring the Concepts of Concentration Addition and Independent Action Using a Linear Low-Effect Mixture Model. *Environmental Toxicology and Chemistry* **2020**, *39* (12), 2552–2559. https://doi.org/10.1002/etc.4868.

(3) Machala, M.; Vondráček, J.; Bláha, L.; Ciganek, M.; Neča, J. Aryl Hydrocarbon Receptor-Mediated Activity of Mutagenic Polycyclic Aromatic Hydrocarbons Determined Using in Vitro Reporter Gene Assay. *Mutation Research/Genetic Toxicology and Environmental Mutagenesis* **2001**, *497* (1), 49–62. https://doi.org/10.1016/S1383-5718(01)00240-6.

(4) Nagy, S. R.; Sanborn, J. R.; Hammock, B. D.; Denison, M. S. Development of a Green Fluorescent Protein-Based Cell Bioassay for the Rapid and Inexpensive Detection and Characterization of Ah Receptor Agonists. *Toxicological Sciences* **2002**, *65* (2), 200–210. https://doi.org/10.1093/toxsci/65.2.200.

(5) Bonati, L.; Motta, S.; Callea, L. The AhR Signaling Mechanism: A Structural Point of View. *Journal of Molecular Biology* **2024**, *436* (3), 168296. https://doi.org/10.1016/j.jmb.2023.168296.

(6) Dai, J.; Dan, W.; Zhang, Y.; Wang, J. Recent Developments on Synthesis and Biological Activities of γ-Carboline. *European Journal of Medicinal Chemistry* **2018**, *157*, 447–461. https://doi.org/10.1016/j.ejmech.2018.08.015.

(7) Li, F.; Li, X.; Liu, X.; Zhang, L.; You, L.; Zhao, J.; Wu, H. Docking and 3D-QSAR Studies on the *Ah* Receptor Binding Affinities of Polychlorinated Biphenyls (PCBs), Dibenzo-*p*-Dioxins (PCDDs) and

Dibenzofurans (PCDFs). *Environmental Toxicology and Pharmacology* **2011**, *32* (3), 478–485. https://doi.org/10.1016/j.etap.2011.09.001.
(8) Rogers, L. J.; Basnakian, A. G.; Orloff, M. S.; Ning, B.; Yao-Borengasser, A.; Raj, V.; Kadlubar, S. 2-Amino-1-Methyl-6-Phenylimidazo(4,5-b) Pyridine (PhIP) Induces Gene Expression Changes in JAK/STAT and MAPK Pathways Related to Inflammation, Diabetes and Cancer. *Nutrition & Metabolism* **2016**, *13* (1), 54. https://doi.org/10.1186/s12986-016-0111-0.
(9) Bin Kanner, Y.; Teng, Q.-X.; Ganoth, A.; Peer, D.; Wang, J.-Q.; Chen, Z.-S.; Tsfadia, Y. Cytotoxicity and Reversal Effect of Sertraline, Fluoxetine, and Citalopram on MRP1- and MRP7-Mediated MDR. *Front. Pharmacol.* **2023**, *14*. https://doi.org/10.3389/fphar.2023.1290255.
(10) Nikolic, I.; Lazovic, A.; Stanisavljevic, I.; Andjelkovic, M.; Popovic, S.; Pavlovic, S.; Jurisevic, M.; Mitrovic, M. Duloxetine's Potential Dual Antitumor and Immunomodulatory Role in Apoptosis and Autophagy Signaling Pathways in Cancer: *In Vitro* and *In Vivo* Evidence. *European Journal of Pharmaceutical Sciences* **2025**, *212*, 107165. https://doi.org/10.1016/j.ejps.2025.107165.
(11) Ferguson, S.; Mesnage, R.; Antoniou, M. N. Cytotoxicity Mechanisms of Eight Major Herbicide Active Ingredients in Comparison to Their Commercial Formulations. *Toxics* **2022**, *10* (11), 711. https://doi.org/10.3390/toxics10110711.
(12) Porcelli, L.; Garofoli, M.; Di Fonte, R.; Fucci, L.; Volpicella, M.; Strippoli, S.; Guida, M.; Azzariti, A. The β-Adrenergic Receptor Antagonist Propranolol Offsets Resistance Mechanisms to Chemotherapeutics in Diverse Sarcoma Subtypes: A Pilot Study. *Sci Rep* **2020**, *10* (1), 10465. https://doi.org/10.1038/s41598-020-67342-6.
(13) Pirozzi, A. V. A.; Stellavato, A.; Schiraldi, C.; Giuliano, M. Herbicide Widespread: The Effects of Pethoxamid on Nonalcoholic Fatty Liver Steatosis In Vitro. *Journal of Toxicology* **2020**, *2020* (1), 7915795. https://doi.org/10.1155/2020/7915795.
(14) El Gendy, M. A. M.; Soshilov, A. A.; Denison, M. S.; El-Kadi, A. O. S. Transcriptional and Posttranslational Inhibition of Dioxin-Mediated Induction of CYP1A1 by Harmine and Harmol. *Toxicology Letters* **2012**, *208* (1), 51–61. https://doi.org/10.1016/j.toxlet.2011.09.030.
(15) El Gendy, M. A. M.; El-Kadi, A. O. S. Harman Induces CYP1A1 Enzyme through an Aryl Hydrocarbon Receptor Mechanism. *Toxicology and Applied Pharmacology* **2010**, *249* (1), 55–64. https://doi.org/10.1016/j.taap.2010.08.014.
(16) Capuzzi, S. J.; Politi, R.; Isayev, O.; Farag, S.; Tropsha, A. QSAR Modeling of Tox21 Challenge Stress Response and Nuclear Receptor Signaling Toxicity Assays. *Front. Environ. Sci.* **2016**, *4*. https://doi.org/10.3389/fenvs.2016.00003.
